# CASMDB: An Open-Source Database of Metabolite Annotation Data for 1D ^1^H NMR-Based Metabolomics

**DOI:** 10.1021/acs.analchem.5c04525

**Published:** 2026-04-21

**Authors:** Morgan W. Hayward, Luca G. Mureddu, Gary S. Thompson, Marie M. Phelan, Edward J. Brooksbank, Geerten W. Vuister

**Affiliations:** 1 Division of Molecular and Cell Biology, Leicester Institute of Structural and Chemical Biology, 98072 University of Leicester, Henry Wellcome Building, Lancaster Road, Leicester LE1 7HN, United Kingdom; 2 School of Natural Sciences, University of Kent, Canterbury CT2 7NZ, United Kingdom; 3 Liverpool Shared Research Facilities (LivSRF) & Institute of Systems, Molecular and Integrative Biology, NMR Centre for Structural Biology, 4591 University of Liverpool, Crown Street, Liverpool L69 7ZB, United Kingdom

## Abstract

In metabolomics analyses, databases are invaluable for the identification of individual metabolites in experimentally collected samples. Publicly available databases for NMR-based metabolomics are unfortunately incomplete with respect to experimental conditions, such as pH, temperature, and NMR field strength, which all affect the observed signals. Moreover, derived NMR annotation parameters, such as peak positions and multiplet patterns, are also often incomplete and contain crucial errors. Hence, these databases are often inadequate for the analyses of experimental samples across a range of conditions, most notably field strength. In this paper, we describe the collection, remediation, and integration of annotation data from the publicly available HMDB, BRMB, and GISSMO NMR metabolomics databases to build the CcpNmr Analysis Simulated Metabolomics Database (CASMDB). CASMDB contains 1932 unique and fully annotated metabolite entries that allow for accurate simulation of spectra at arbitrary field strengths. CASMDB can be downloaded as a standalone, versioned repository from GitHub and easily augmented with new entries. CASMDB underpins the visualizing of experimental and simulated metabolite references and allows for 1D ^1^H NMR-based metabolomics studies.

## Graphical abstract

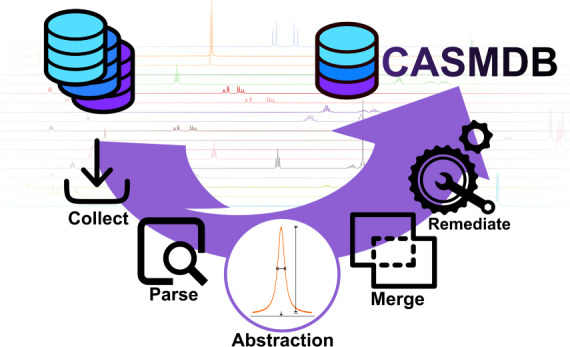

Metabolomics is the field of global systems biology concerned with small molecules, i.e., with molecular mass <1.5 kDa, that are substrates or products of metabolism, also known as metabolites. The collection of metabolites in tissue, fluids, or biological samples in general constitute the so-called metabolic profile.^1^ Metabolomics studies typically aim to identify biomarkers of metabolic processes or disease and have applications in a wide variety of research interests including pharmaceuticals,^2^ personalized medicine,^3^ nutrition,^4^ microbiology,^5^ agriculture,^6^ marine biology,^7^ and toxicology.^8^

The two premiere experimental techniques used in metabolomics analyses are mass spectrometry (MS) and nuclear magnetic resonance (NMR) with each technique associated with its own advantages. MS has the greater sensitivity of the two techniques, whereas NMR affords the inference of invaluable chemical knowledge albeit at a lower intrinsic sensitivity.^9^ In addition, NMR-based metabolomics is highly automatable and reproducible and can be used for the observation and comparison of whole metabolite profiles without comprehensive (*a priori*) knowledge of the metabolites in the sample, also known as untargeted metabolomics.^9^

The comprehensive chemical information provided by NMR intrinsically adds complexity to the data as each metabolite is associated with a characteristic set of peaks in the spectrum described by chemical shifts, and their associated coupling patterns or “multiplets” that derive from the specific chemical environment for each atom. In the same fashion that a metabolite signature is the collective signal of all its peaks, the spectra obtained in NMR-based metabolomics are the concentration weighted sum of the signatures of all metabolites in the sample and can often comprise hundreds to thousands of peaks. As such, high-quality databases of metabolite spectra, whether from publicly available sources or otherwise, are invaluable to NMR-based metabolomics researchers.^10^

Limitations in these resources arises as NMR metabolite signatures are specific to the instrumentation field strength. The two most well-known publicly accessible databases for NMR-based metabolomics are the Human Metabolome Data Base (HMDB)^11^ and the Biological Magnetic Resonance Data Bank (BMRB)^12^ (Table 1) containing entries collated in the past 20 years. Although HMDB and BMRB contain 1000+ spectra, not all data is scalable for the higher field strengths prevalent in current (and future) studies. The HMDB contains entries for >240,000 metabolites and contains extensive biological metadata including metabolite chemical details, typical concentrations in biological samples and ontological classification. However, HMDB is primarily focused on MS spectra rather than NMR and only contains entries for 1372 unique NMR metabolites. The BMRB is a biological NMR-specific database containing >972 unique experimentally supported entries, making it particularly reliable for NMR-based researchers. However, unfortunately it lacks the extensive metabolite metadata of the HMDB.

**Table 1:** Overview of Commonly Used Freely Available and Commercial Databases for Metabolomics

	HMDB	BMRB	GISSMO	Chenomx	KnowItAll
number of metabolite entries	248,047 (1372)^a^	2496 (972)^a^	658 (581)^a^	338 (338)^a^	157,465 (unknown)^a^
modal frequency or frequency range (MHz)	600	500	N/A	500–800	unspecified
main chemical shifts reference used	DSS	DSS	DSS	DSS	unspecified
database type	experimental and theoretical	experimental and theoretical	simulated	simulated	experimental
species specific	yes	no	no	no	no
NMR specific	no	yes	yes	yes	yes
metabolomics specific	yes	no	yes	yes	no
freely available	yes	yes	yes	no	no
reference	^11^	^12^	^15^	^13^	^14^
link	https://hmdb.ca	https://bmrb.io	https://gissmo.bmrb.io	https://www.chenomx.com/libraries/	https://sciencesolutions.wiley.com/software/

a Unique metabolite entries with sufficient NMR data in brackets.

Scalable data exists in commercial metabolomics databases (cf. Table 1) such as the proprietary Chenomx Library; however, these databases are structured around specific biofluids (urine, plasma, etc.). This well-known commercial NMR-based metabolomics database is coupled with the Chenomx NMR Suite,^13^ which contains annotated spectra for 336 compounds at a range of spectrometer frequencies and pH levels. Alternatively, commercial analytical chemistry NMR databases such as the KnowItAll NMR Spectral Database Collection^14^ contain vast amounts of NMR spectra, which include metabolites. While the commercial databases tend to be more consistent in terms of sample conditions and annotation compared to the publicly available counterparts, they require costly subscriptions. Moreover, their closed-source nature often results in reduced usage flexibility, making them even more difficult to employ for academic research projects.

Given that metabolite NMR spectra can vary depending on the sample and spectral conditions, such as pH, solvent, and field strength, simulating spectra through field independent calculation of the full Hamiltonian, i.e., spin-system-based simulations, offers an attractive alternative. The Guided Ideographic Spin System Model Optimization library (GISSMO)^15^ is an example of one such effort; it contains the information on metabolite spectra abstracted as spin system matrices (SSMs) that encode the chemical shift and coupling constants of the protons in a given metabolite. Peak lists are generated from these SSMs and are completely field independent. A large subset of the GISSMO simulated spectra is effectively used in deconvolution Web servers such as COLMAR1d.^16^ Notably, simulating spectra this way becomes significantly more demanding for larger metabolites as the simulation algorithm has an exponential time complexity.

We have developed a collated and curated database called the CcpNmr AnalysisMetabolomics Database (CASMDB) for simulated reference spectra from the HMDB, BMRB, and GISSMO open-access data sources. The freely available CASMDB allows for simulation of metabolite reference spectra independent of magnetic field strength as well as independent manipulation of peak multiplets for improved accuracy in metabolite matching. The CASMDB abstracted data are stored using the NMR Exchange Format (NEF)^17^ to yield a portable and scalable resource combining accessibility and compatibility for integration with any established NMR analysis software. An example of such software is the CcpNmr Analysis suite^18,19^ for biomolecular NMR.

## Methods

### Data Sources

The annotation data, i.e., spectral peak lists and metabolite meta data, were downloaded from the HMDB (https://hmdb.ca), BMRB (https://bmrb.io/metabolomics), and GISSMO (https://gissmo.bmrb.io) repositories for all available experimental entries in various formats (*vide infra*). Entries that did not contain experimental data or were not completely annotated were labeled appropriately so that only complete data sets were analyzed and made available. Theoretical data sets, i.e., peak lists generated from chemical structure alone without measurement of experimental data, were not included in the database. Table 1 gives an overview of relevant database statistics.

HMDB annotation data for NMR-based metabolomics was downloaded from https://hmdb.ca/downloads for processing. All metabolite biological and chemical data were available as a single large Extensible Markup Language (XML) file, whereas sample and spectral data, including peak lists, were available in in a variety of formats: XML (*n* = 2924), text (TXT) (*n* = 1002), and/or NMR Markup Language (nmrML) (*n* = 773). Where appropriate, files were regularized and refined to allow automatic parsing (Table S1).

Metabolite data were extracted automatically prioritising nmrML files due to their inclusion of supporting spectrum intensity arrays and multiplet assignment, followed by XML files and then TXT to supplement sample and spectrum data whenever the nmrML data alone proved insufficient. When matching data across multiple files, the HMDB accession numbers were used to match metabolites to their annotation data and HMDB experiment numbers were used to match sample, spectrum, and peak data.

BMRB experimental data were retrieved in bulk from https://bmrb.io/metabolomics in Self-Defining Text Archive and Retrieval (STAR) files (*n* = 2496) defined using the BMRB V3 NMRS-STAR dictionary. The original data were parsed using the official BMRB API python package^20^ to retrieve metabolite, sample, and spectral data, including peak data; however, no multiplet assignments were available. The available metabolite meta data were limited to name, description, chemical formula, average molecular weight, Simplified Molecular-Input Line-Entry System (SMILES), and International Chemical Identifier number (InChI). Of the 2496 files, 1414 files were excluded due to insufficient peak annotation data (Table S1).

Peak width data were unavailable for most HMDB and BMRB entries, in which case a placeholder value of 1 Hz was used.

GISSMO spin system matrices were downloaded from https://gissmo.bmrb.io in XML format (*n* = 659). Spin system matrices were extracted automatically and stored in tabular format; proton chemical shifts were stored as an equivalent to multiplet chemical shift data and coupling constants in a unique table, which contains the coupling values and the proton/multiplet identifiers for coupling assignment. As GISSMO entries are an extension of BMRB entries, metabolite, sample, and spectral data were inferred from the BMRB data with the same accession numbers. No peak data were available from the GISSMO files due to the nature of the data.

### Remediation Pipeline

To ensure that the NMR spectrum simulations are representative, they were compared against the experimental spectra wherever available in the source database entry. Simulated spectra were generated to exactly match the experimental spectra with respect to sweep width and number of data points. Simulation accuracy was assessed using cosine similarity (eq 1), which scores between 1 for a perfect match and −1 for a perfect inverted result, and proved suitable sensitive to variations in position and line width (cf. Figure S1).

$$S=1-\left(\frac{\mathrm{A⃗}\cdot \mathrm{B⃗}}{∥\mathrm{A⃗}∥∥\mathrm{B⃗}∥}\right)$$ 1

where *A→* and *B→* denote the 1D vectors comprised by the data arrays of the two spectra to be compared.

Overall similarity scores were calculated from the mean of the similarity scores of spectrum subsections containing simulated peak data, i.e., the regions of interest, to prevent solvent and reference peaks interfering with the similarity scores. A total similarity score of 0.9 was used as a threshold of sufficient correlation of the simulated spectrum with the measured. A score of 0.9 is considered an acceptable match score as this corresponds to a width ratio between query peak and match peak of 2.5 and excludes peaks with signal-to-noise less than 10, i.e., the accepted limit of quantification, as well as false positives derived from inclusion of additional query peaks (Figure S1). All simulations with similarity scores below 0.9 were automatically adjusted for overall peak widths, alignments, and solvent suppression. Any simulations that could not be automatically modified to reach a similarity score of ≥0.9 were visually assessed and remediated manually. Simulation matches with scores of *exactly* 1.0 were also manually inspected to ensure scores were verified to experimentally derived data.

Simulations were remediated by updating peak- and multiplet data in peak-list-based simulations and by adding spin–spin couplings in spin-system-based simulations. Appropriate peak and multiplet data were gathered by peak picking and multiplet assignment of the associated experimental spectra in the CcpNmr AnalysisMetabolomics program, which is part of the CcpNmr software suite.^18,19^ All remediations were made with reference to the chemical structure of the metabolite to ensure appropriate multiplet assignment. All experimental spectra were imported into AnalysisMetabolomics using their native binary formats and also converted and saved in NMR Hierarchical Data Format files (NDF5), which are based on the HDF5 standard (to be published). These NDF5 files contain the processed spectral data, including any remediation such as phase correction and chemical shift reference alignments and are available to download in bulk from the Github page (https://github.com/ccpnmr/CASMDB) for a consistent collection of remediated experimental data.

### CASMDB Database Consolidated Format

All data processed from the respective databases was saved into a tabular format for compact storage and efficient access during remediation. The finalized database was converted to a directory of NMR Exchange Format (NEF)^17^ files, with each NEF file documenting the data for a unique metabolite to allow ease of access and filtering both locally and online. The database was made with a structure that reflects the biological relationships of the data as well as the data structures of the CcpNmr AnalysisMetabolomics program to allow easy access and deposition to and from the database (Figure 1). Due to the abundance of metadata from the HMDB, the core metabolites table and the metabolite metadata tables were structured similarly to the HMDB metabocards. However, the ontology data from these metabocards is stored in a single table in CASMDB, as opposed to the hierarchical format in the HMDB. Metabolites from the BMRB and GISSMO repositories were matched to HMDB entries via International Chemical Identifier (InChI) and stored under the same entry in the metabolites table to avoid duplicates. Unique CASMDB IDs were created for each table to ensure distinct data relationships. Database origin accession numbers were stored in the most appropriate tables, e.g., HMDB accessions are unique and unambiguous at the metabolite level whereas BMRB accessions are ambiguous at the metabolite level; therefore, HMDB and BMRB accessions were stored in metabolite and sample tables, respectively.

**Figure 1:**
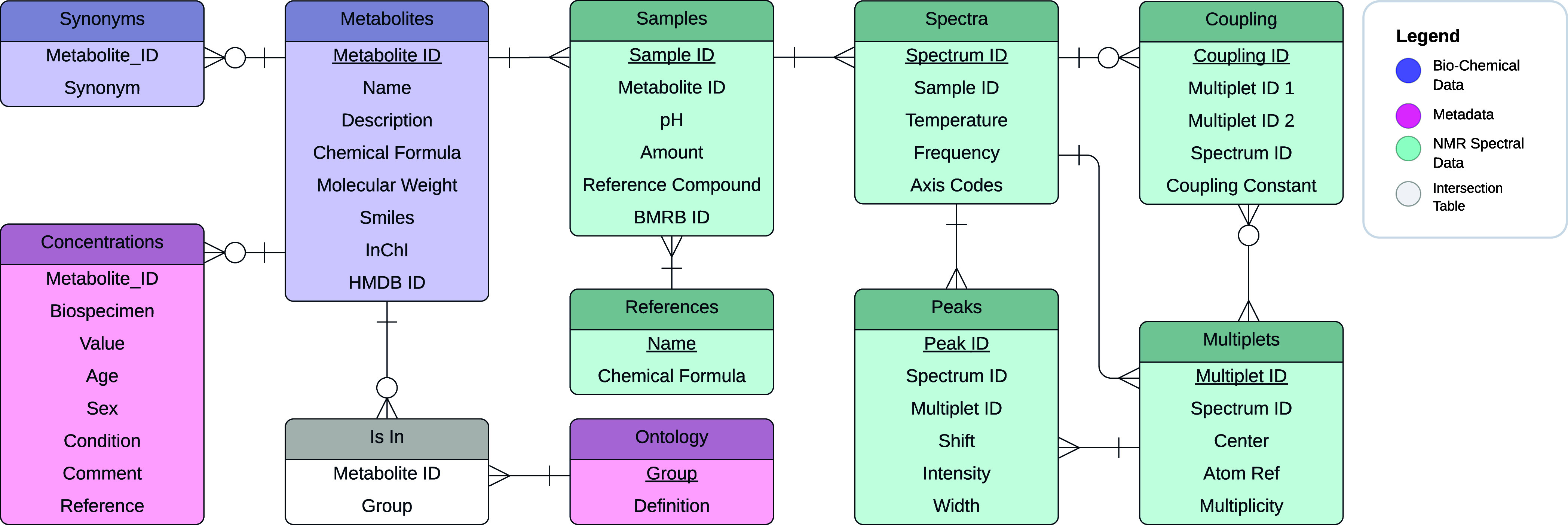
Entity Relationship (ER) diagram depicting the structure of the CcpNmr Metabolomics Database and the relationships between the various tables. Colors indicate classification of the data contained in the table: metabolite data (purple), metabolite meta data (pink), and NMR data (teal). Connectors indicate the relationships between the tables, all of which are many-to-one (⪫—+—) or optional-many-to-one (⪫O—+—), e.g., a metabolite entry is linked to many samples, but each sample is linked to one metabolite. The Ontology entity is the only exception as multiple metabolites can be in multiple biological samples, hence the inclusion of the “Is In” table to create a many-to-many relationship.

The CASMDB is available as a versioned database from the CcpNmr Github repository at https://github.com/ccpnmr/CASMDB. We encourage user contributions, through a pull request, which will be followed by a review of the data quality, as detailed on the repository, prior to acceptance. Ideally, a user contribution would be paired with reference data deposited in a public repository, such as the HMDB, BMRB, or MetaboLights,^21^ offering independent curation and verification. An unverified contribution would necessitate a more stringent review.

### Spectrum Simulation and Validation

Spectrum simulations were created using a modified version of the publicly available nmrsim^22^ python package for simulating the NMR spectra of spin-1/2 nuclei. Spectra simulating functions from the nmrsim package were modified to allow width assignment to individual peaks. Simulated spectra are created from the metabolite data by transforming peak chemical shifts, heights, and widths into Lorentzian distributions, which are summed to create the spectrum intensity arrays. For entries without peak lists, i.e., GISSMO entries, peak lists are created from multiplet chemical shift and coupling constants via the nmrsim quantum mechanics (qm) functions. Simulations were visualized using the CcpNmr Analysis software.^18,19^

An experimental standard mixture was prepared at three pH levels containing 22 commonly observed metabolites (cf. Table S2) encompassing complex spin systems, p*K*_a_’s, and strong couplings representing the typical challenges of metabolomics samples. All mixtures were prepared in 99.9% ^2^H_2_O with 100 μM trimethylsilyl propionate (TSP; 2,2,3,3-d4 selectively deuterated) and 100 mM sodium phosphate at either pH 7.2, 7.4, or 7.6. The spectra of these samples were acquired at three field strengths of 600, 700, and 800 MHz under uniform conditions, pulse sequences, and parameters (Table S3 and MetaboLights data set MTBLS6263, https://www.ebi.ac.uk/metabolights/MTBLS6263). In most cases, the BMRB and HMDB data at 700 or 800 MHz was not available and multiplet matching analysis using the similarity scoring (eq 1) was limited to the 600 MHz data set only (Table S4). Overall matches for 16 metabolites using BMRB, 3 HMDB, and 21 GISSMO matches were possible. Individual multiplet analysis was performed on GISSMO remediations only due to the coverage at multiple fields.

Experimental standard mixture spectra were compared to CASMDB-simulated references and where applicable (equivalent field strength, pH) the original reference data using similarity scoring (eq 1), which was employed in remediation. To account for the offset between the standard used (TSP) and the database reference standard (DSS),^23^ as well as any other experimental offsets, simulated spectra were aligned to the multiplet at the lowermost end of the spectrum. For both alignments, a separate similarity score was calculated per multiplet.

All data and scripts used to create tables and figures can be accessed at the Zenodo 10.5281/zenodo.19468941.

## Results and Discussion

Overall, 1932 unique metabolite entries, as determined by their InChI codes, were included in CASMDB, with 3329 samples, 3333 spectra, 24,021 multiplets, and 71,485 peaks (Table 2). The HMDB was the highest contributor of metabolite entries of the three databases queried, and it is also the only contributor for ontology and concentration data. Although the relationship from sample to spectrum is considered as one-to-many, i.e., there can be multiple spectra recorded for a single sample, this was only the case in four samples with the vast majority of entries only having one 1D ^1^H spectrum for each sample. No multiplets were extracted from the BMRB entries as peak to atom assignment was not possible from the current BMRB NMR-STAR files. No peak data were imported from GISSMO entries due to the nature of the data.

**Table 2:** Summary of the Content of CASMDB

	HMDB	BMRB	GISSMO	CASMDB
metabolites^a^	1372	972	581	1932
synonyms	40,031	12,865	0	52,566
ontology	53,731	0	0	53,731
concentrations	24,250	0	0	24,250
samples	1471	1200	658	3329
spectra	1471	1204	658	3333
multiplets	19,201	0	4820	24,021
peaks	38,931	32,554	0	71,485

a The total for metabolites and synonyms is not equal to the sum of the corresponding numbers of the individual databases, as the databases are not mutually exclusive.

Metabolite distribution across the three original databases was greater than expected with only 458 of the 1932 metabolites shared between the HMDB and the BMRB repositories, when considering the GISSMO entries as extensions of the BMRB (Figure S2). Even though GISSMO entries were developed from BMRB data, data for 46 metabolites extracted from the GISSMO repository were not present in BMRB. This is due to the many exclusions required for the initial parsing of the BMRB data (cf. Table S1) due to missing peak data. The data retrieved from the HMDB was the most exclusive with 914 metabolites not found in either of the BMRB or GISSMO databases.

There was a range of sample and spectral conditions for the data from each source, with the HMDB data typically showing the largest variation (Figure S3). Most samples were obtained at a pH between 6.5 and 7.5, with HMDB spectra typically recorded at pH 7.0 (*n* = 855) and BMRB spectra at pH 7.4 (*n* = 691). The full range of pH values was between 1 and 12 for HMDB samples and between 7 and 12 for BMRB samples. There were 1208 samples with missing pH values. Of the 438 metabolites shared between the HMDB and BMRB/GISSMO, 306 metabolites in CASMDB have spectra collected at more than one pH value as a result of the merging of the original databases. Sample references were most commonly 2,2-dimethyl-2-silapentane-5-sulfonate sodium salt (DSS) or tetramethyl silane (TMS), with BMRB samples exclusively using one of these references. A minority of HMDB samples used CHCl_3_ or trimethylsilyl propionate sodium salt (TSP) as the reference compound and six samples did not have a reported reference compound. The spectrometer frequency ranges for the HMDB spectra were between 90 and 700 MHz, whereas the BMRB spectra, and therefore also the GISSMO data, were exclusively recorded at 400, 500, and 600 MHz. Fifty-one HMDB entries had missing data for the spectrometer frequency, whereas only one BMRB spectrum (for entry bmse000060) was missing this data. Temperature data are not displayed in Figure S3 as all spectral data were listed as being recorded at 298 K.

### Remediation of Database Entries

Simulated spectra could be recreated from peak list data, to act as a means of identifying potential issues and for relative quantification. The HMDB and BMRB simulations were made from annotated peak lists whereas GISSMO simulations generate peak lists, which are based on *J*-coupling interactions. The differences between these two approaches can be seen in the examples where the GISSMO simulation recapitulates the smaller peaks at the edges of the multiplet that could be lost in the experimental noise.

The requirement for remediation and suitability of the adjustments made was established via use of cosine similarity scoring (eq 1). While not an innovative metric, it is potentially underutilized in NMR peak matching, offering a metric that is both sensitive to chemical shift and tolerant to line width (cf. Figure S1).

During the CASMDB remediation (Figure 2), a total of 1469 simulated spectra failed to meet the similarity accuracy threshold of 0.9 and underwent remediation. For 959 spectra, automatic remediation by optimizing line width and position was sufficient to meet the threshold. A further 510 spectra required manual remediation to peak positions and/or heights, multiplet patterns, or *J*-couplings (Figures 2 and 3A). Examples of the various manual remediations are shown in Figure S4.

**Figure 2:**
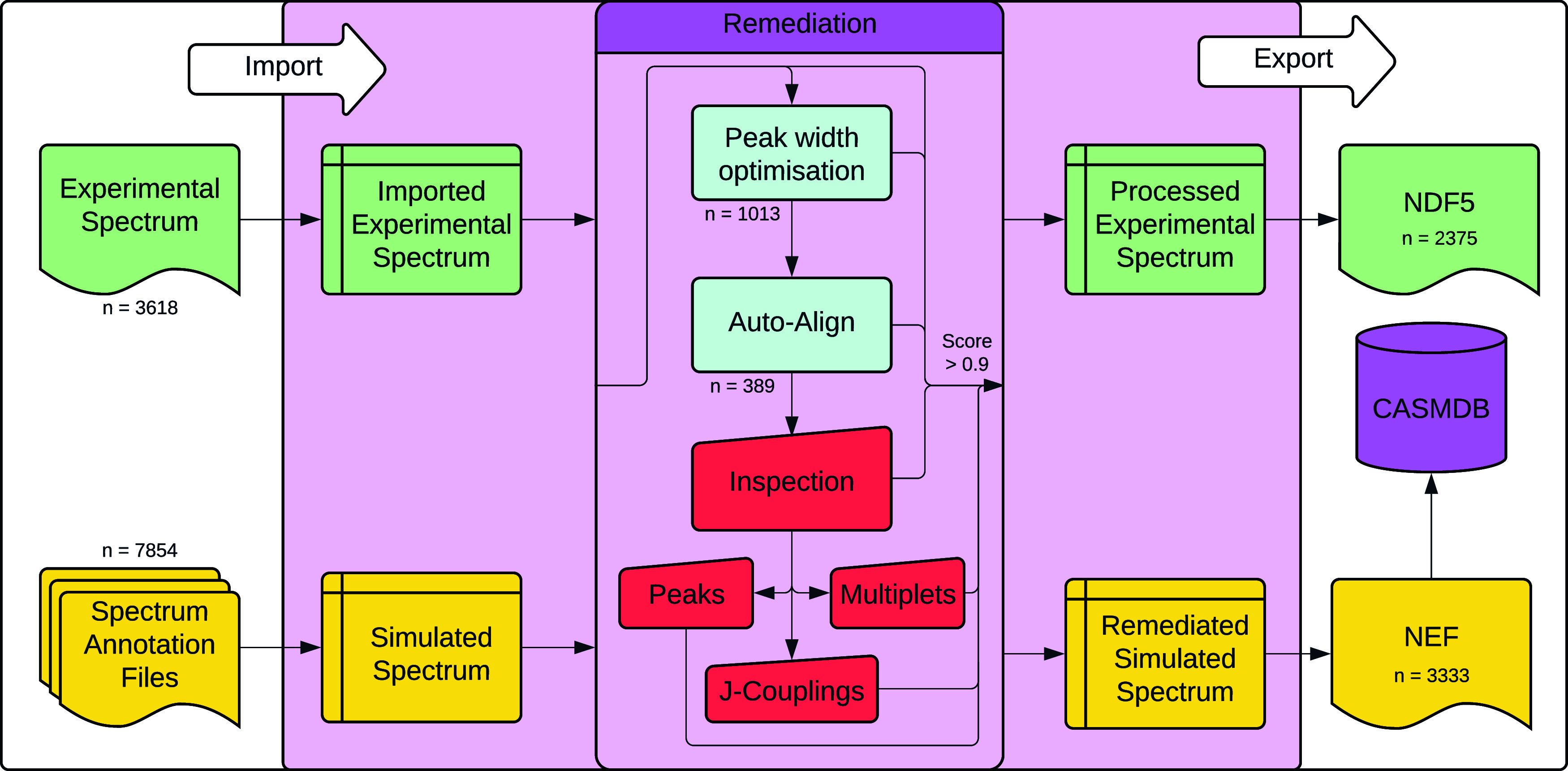
Remediation pipeline. Number of spectra improved at each remediation step indicated within the process box with some spectra requiring multiple steps to reach the acceptance threshold of 0.9.

**Figure 3:**
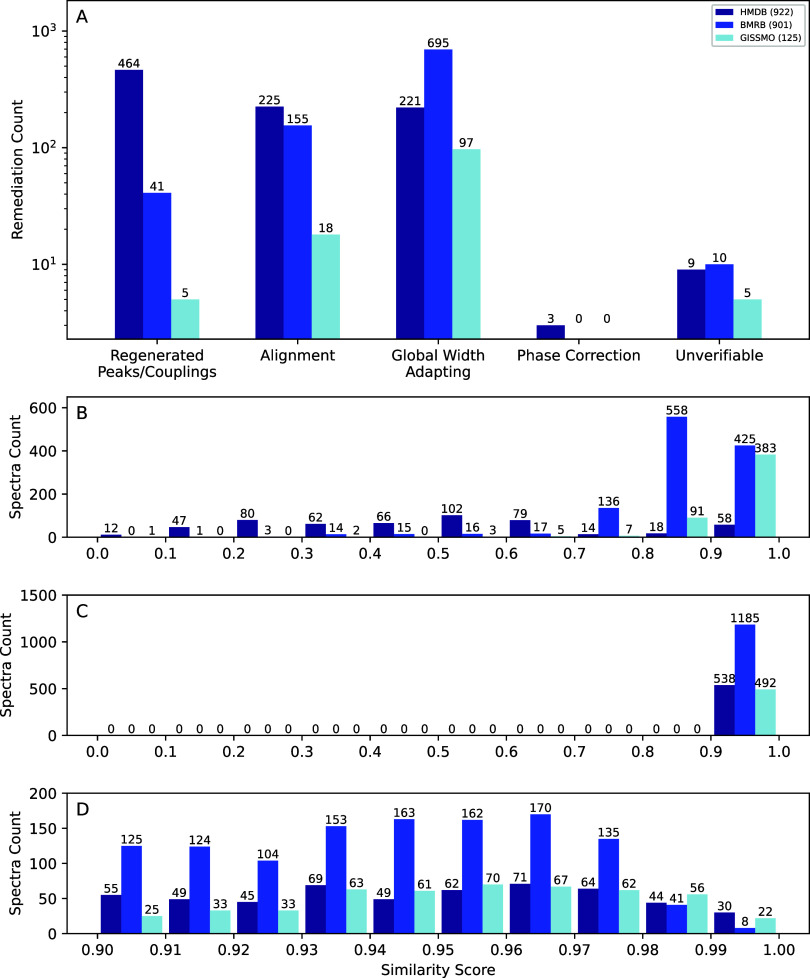
Summary of the remediation of CASMDB. (A) Bar chart of the remediation categories using logarithmic scale for count. (B–D) Histograms of the accuracy scores using cosine similarity before (B) and after (C, D) remediations. (D) is an expanded view of (C). Count number refers to the number of spectra that were remediated. The database origin is indicated by color coding.

Most manual remediation was made to the HMDB data, most often as a result of low precision in peak positions. Many HMDB spectra were recorded at a precision of +–0.005 ppm, which is typically not enough to recreate multiplet patterns with sufficient precision, as many peaks become indistinguishable from each other. The primary remediation requirement for BMRB data was to increase the overall peak width, typically to 3 Hz. Only five entries from the GISSMO database required adding of additional couplings, with most simulations being highly representative of the associated experimental data from the BMRB. It is noteworthy that GISSMO-simulated spectra will lack common features of experimental spectra, such as noise, artifacts, reference peaks, and water/solvent signals. Such simulations also do not include any errors in the line shape of the spectra, such as those resulting from poor phasing or non-Lorentzian line shapes due to poor shimming or apodization. A small subset of 24 poorly matching simulations could not be remediated due to either low quality or incorrect spectra in the experimental file. Any simulation that could not be verified, including simulations without any associated experimental spectra, are still included in the database but marked as unverified. Table S1 shows typical examples of remediation and exclusion. Remediation improved 61% of spectral simulations (Figure 3B–D) with 1% redefined as unverifiable. Remediation and manual inspection of low scoring spectra allowed for identification of false negatives due to issues with original experimental reference data such as misalignment. Perfect scores of 1.0 were also indicative of “cleaned” or experimental data with an absence of noise and were marked as “unverified” in CASMDB. Other sources of false positives, such as inappropriate multiplet definitions or simulations of nonidentical derivative molecules of metabolites that are similar enough to the associated experimental spectrum to pass the threshold, could persist. As such, the community is encouraged to report potential errors at the GitHub repository for iterative improvement of the database.

### Validation Using a Standard Mixture

Experimental standard mixtures were prepared at three pH values and spectra were recorded at 600, 700, and 800 MHz spectrometers (Figure 4A). The data were evaluated for how well they matched to the original reference data sets and to the remediated CASMDB entries to validate the improvement in identifying metabolites. Strong coupling, where chemical shift differences are on the same scale as *J*-couplings, presents a common issue in small-molecule NMR data. Figure 4B–D displays three metabolite regions at three field strengths, with the phenomenon particularly evident for the citric acid doublets (Figure 4B), which exist as a coupled pair of doublets with ∼60 Hz separation. Note that the downfield doublet overlaps with additional other signals. Figure 4E–G clearly shows the effect of pH, particularly for the upfield citric acid doublet (Figure 4E) leading to small but significant shifts (∼0.01 ppm) of the resonance positions, deteriorating the match with the unadjusted original reference data. The CASMDB-derived spectra at the three fields (Figure 4H–J) clearly shows the variable strong-coupling patterns and the poor match to the experimental data if the field strength is not considered. We assessed the matching of the CASMDB simulations against the spectra also provided by GISSMO. Figure 4K–M show the similarity scores for citric acid and l-valine multiplets, which improved from <0.5 to >0.9.

**Figure 4:**
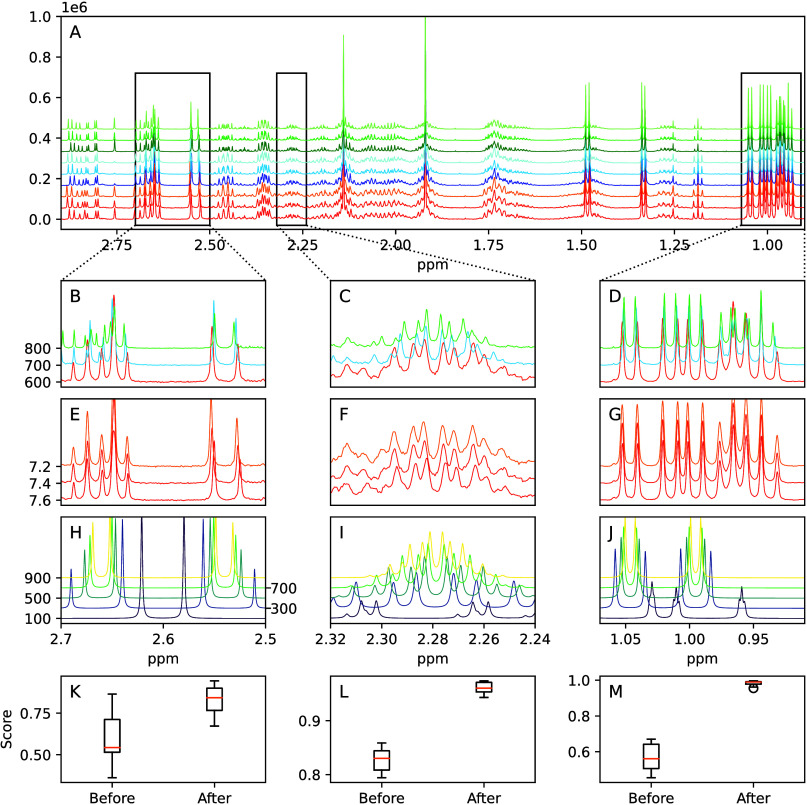
Experimental standard mixture evaluated at three field strengths and three pH values to demonstrate field-transferable identification. (A) Overlay of all nine experimental standard mixtures. (B–D) Zoomed views of the three regions in (A) showing spectral effects of different field strengths (indicated as MHz). (E–G) Zoomed views of the same three regions showing the spectral effects of changes between pH 7.2 and 7.6. (H–J) Zoomed views of the same three regions now showing spin-system-based simulations calculated from 100 to 900 MHz. (H) Two doublets of citric acid. (I) Doublet of quartets of quartets of the H^ß^ of l-valine. (J) Two doublets of the methyl- groups of l-valine. (K–M) Box plots of the similarity scores (eq 1) of GISSMO-simulated spectra before and after introduction into the CASMDB for the multiplets in (H–J).

Table S4 contains the full collection of similarity scores for 85 multiplets of 22 metabolites for the 600 MHz mixture standards spectra. Figure S5 displays the overview derived from these data showing an average increase in similarity score of 0.177 and 0.148 for HMDB and GISSMO, respectively. However, the BMRB data showed a minor decrease of 0.012.

Table 5A–C summarizes the similarity score between the GISSMO and the CASMDB reference simulations at three field strengths and three pH values. Figure S6 displays these scores as box plots. In all cases, usage of CASMDB show a convergence of scores toward a similarity score of 1.0.

### Database Structure

CASMDB was designed with minimalism and simplicity in mind, as it was not intended to function as a storage mechanism for the experimental spectral data akin to HMDB or BMRB. Instead, it is intended to function as a portable and scalable source for generating metabolite standard spectra for identification and quantification purposes. The collation, remediation, and abstraction of the source data into CASMDB now allows for the flexible, real-time visualization of 1932 metabolites, increases of 41, 99, and 232% relative to the data obtained from the individual HMDB, BMRB, and GISSMO databases, respectively (Table 2).

The core of the database comprises the data required to recreate spectra, but each CASMDB entry also contains the necessary IDs and accession numbers to locate the original source. This simplicity also ensures that the database is relatively compact at 62.2 MB expanded or 11.9 MB when compressed, allowing easy downloading and thus rapid access to metabolite overlays. The use of peak lists and spin system matrices over spectra, which contain large intensity and position arrays, also contributes to the compactness of the database, as a spectrum can be recreated from tens to hundreds of peak values at any required resolution. A simpler database format is also advantageous for user interaction, as it allows quick and easy deposition of custom metabolite entries.

The database also contains tables for crucial metabolite metadata, which, while it is not strictly needed for accurate reference overlays, was included for increased usability. Specifically, it allows for quick reference to the biological context of the molecule without having to trace back the entry to its respective database webpage; this also allows the database to operate without an Internet connection. Moreover, it allows for additional annotations, such as ontologies, by the user that might currently be absent from the data. We envision such information to ultimately feed back into CASMDB to enhance its functionality for all users. The Github Web site (https://github.com/ccpnmr/CASMDB) details the process of addition or modification of the CASMDB.

### Database Content

The total of 1932 distinct metabolites can be considered large from a practical perspective, as most NMR-based metabolomics studies will typically identify/quantify 50 to 200 metabolites, which is an order of magnitude less than the metabolites contained within CASMDB. However, the 1932 metabolites available for NMR analysis are still surprisingly few considering the total number of metabolites available in the source databases, with the HMDB alone containing entries for >240,000 distinct entries. This highlights an alarmingly large issue of missing annotated data in NMR-based metabolomics databases likely due to many contributing factors. We suspect that one such factor may simply be the lack of data deposition; the HMDB is substantially more focused toward MS-based metabolomics, with many entries containing dozens of MS spectra but often only one or no NMR spectra. Although it may be reasonable to expect a greater number of depositions for the technique with greater sensitivity, recent improvements in NMR hardware and the improving access to high-field NMR spectrometry means resolution in NMR-based metabolomics is ever improving and database references will need to be expanded accordingly. Notably, CASMDB can easily be augmented by its users with new entries, and consequently also funneling into the repositories of experimental data, such as HMDB and BMRB, or *vice versa.*

Another factor for missing annotation data is that public databases were likely never designed for simulation purposes but rather for searching and matching to peak lists. This is most evident with the BMRB database, which creates its peak lists by automated peak picking of experimental spectra, thus allowing users to search for hits via peak lists. Furthermore, over half of the BMRB entries had to be excluded due to insufficient peak data (Table S1), mostly due to lack of peak intensities that are not necessary for searching by peak list, but vital for creating simulations. CASMDB remedies this issue and is fully searchable by any attribute of the spectrum annotation data.

Another potential issue with the scope of the database content concerns the sample and spectral conditions. Ideally, we would have every metabolite recorded at several relevant conditions, typically varying pH and temperature, but this is rarely the case as it requires extra resources. Spectrum differences caused by minor variations in pH and temperature (cf. Figure 4E–G) can often be accounted for by allowing some flexibility in the chemical shifts of multiplets, and even spectrometer frequency can be extrapolated by rescaling peak positions relative to the multiplet they are assigned to. These adaptations are only possible if peak to atom assignments are present, which is the case in the HMDB and GISSMO entries but not in the BMRB entries. The BMRB entries instead account for the condition differences present, i.e., spectrometer frequency differences, by having more spectra for the same metabolite, as shown by the higher metabolite to spectrum ratio in the BMRB entries. CASMDB has collated all this information into one resource and solves the most pressing of these issues, i.e., the field dependence of the data, using spectrum simulations (*vide infra*) while also allowing for lightweight peak-list-based simulations for more complex metabolites, to be provided in the same location and file format.

### Spectrum Simulations

Visual inspection of the spectra show that those created from peak lists are suitable as references for identification and quantification purposes only in the case of weak-coupling spin–spin splitting patterns. Furthermore, creating simulated spectra via peak picking is most accurate where peak heights and positions are well resolved; otherwise, the simulation accuracy is reduced, particularly in dense regions of the spectra where the peaks overlap considerably and where the signal-to-noise ratio is low. Under these circumstances, multiple overlapping signals can contribute to the shape of each other and/or as a single peak, often increasing the total height and width of the peak/s and rendering peak picking alone insufficient as a strategy for simulated spectrum data collection. Under these circumstances, proper signal deconvolution using other technologies is necessary and the CASMDB-based approach can provide for the required data underpinning any deconvolution algorithm.

Spin-system-based simulated spectra do not suffer from the various problems outlined above, as they construct the data from first-principles, essentially starting with a single signal for each proton and distributing that signal throughout the splitting pattern resulting from the *J*-coupling interactions, with all effects including those of field strength, and strong and weak couplings properly accounted for. The absence of noise and artifacts further improves the reliable identification of the metabolites (cf. Figures S1 and S4). Nevertheless, there are limitations for spin system simulations as well. First, they require accurate measurements for multiplet chemical shift and *J*-coupling values, something that can be particularly difficult in more complex spectra and take longer to record and analyze compared to automated peak picking. Hence, CASMDB contains a carefully curated set of such data. Second, for medium to large metabolites, spin system simulations take significantly longer to generate computationally, compared to spectra produced from peak lists only, as the algorithm to produce peak lists from spin system matrices unfortunately has a nonlinear time complexity with respect to the number of spins in the simulation—roughly O­(N^3^) for simple simulations, thus limiting simulations to approximately 12–15 spins without segmenting spin systems. Using segmented approximations, it is possible to get to much larger systems.^24^ Consequently, the current basic implementation of spin system simulations is best suited to relatively small metabolites, i.e., those with 10 or fewer coupled protons, or else they can currently not easily be manipulated in real time during user interaction.

### Database Accessibility and Development

No database is complete upon its creation, and CASMDB is no exception. CASMDB will need updating, expanding, and maintaining. External users are encouraged to augment CASMDB as the NEF framework is fully defined for the preparation of new metabolite entries (https://github.com/ccpnmr/CASMDB). Additional expansion efforts include gathering additional metabolite spectra from other publicly available databases. Good candidates include the Natural Product Magnetic Resonance Database (NPMRD),^25^ an NMR-specific database of small molecules developed by the Wishart Lab in a similar style to the HMDB, although not specific to human metabolites. A second database is the Birmingham Metabolite Library (BML),^26^ a relatively small but particularly thorough database of 1D ^1^H and 2D *J*-resolved NMR spectra of metabolites under a range of pH values and acquisition times. As CASMDB can easily be expanded, its coverage can be enlarged to also include a variety of scientific areas such as nonhuman biological systems, which currently are under-represented with respect to metabolite standards. Efforts to accomplish such expansions are currently being pursued. Furthermore, in the case of duplicate entries, spectra can be available at multiple conditions. This data can then be analyzed to yield information regarding the effects of experimental parameters, such as pH or temperature, or to place limits on the confidence intervals for data measured across independent replicates.

An alternative to simply collecting and annotating more spectra is presented here where we develop and expand the use of spin systems and spectral simulations in NMR-based metabolomics. Simulations are exceptionally flexible by nature, and so, theoretically, if annotated in enough detail, spectra only need to be recorded once to extract the parameters to cover all sample and spectral conditions. With modern NMR spectrum viewing software, proper annotation of spectra is easier than ever, including the creation and refinement of simulations from spin system matrices. We have developed a CcpNmr AnalysisMetabolomics module (freely available for academic usage from https://ccpn.ac.uk/software/downloads/) for real-time creation of spin system matrices from measured experimental metabolite standards, thus allowing user creation of spin-system based simulations and entry into CASMDB.

### Limitations

The CASMDB offers an enhanced database for metabolite matching in NMR metabolomics. However, there are limitations inherent in any database particularly in a field applied to such a wide range of biofluids and materials. This diversity can lead to metabolite peak chemical shifts changes, which is compounded by changes resulting from acquisition at different temperatures and by samples with different buffer conditions. While the CASMDB has been compared to an experimental standard mixture at three pH values and field strengths, its strength lies in modeling intracellular metabolite extracts, which vary in both composition and matrices of macromolecular components such as albumins. However, it is anticipated that while its metabolites would still be matched within proteinaceous biofluids such as blood plasma, blood sera, and those with extreme variation in ionic strength such as urine, the exact scoring would vary from those without extra components.

## Conclusions

CASMDB functions as a free and easy-access reference database of simulating representative metabolite spectra for NMR-based metabolomics research. It has been built with the explicit purpose of spectral simulations and consequently has a concise structure dedicated to storing only the necessary parameters to simulate metabolite 1D ^1^H NMR spectra. These data are stored in the FAIR NEF format,^17^ allowing for retrieval and usage by multiple software packages. We expect and hope that the database will grow in due course, as well as receive additional tools for spectral manipulation and custom reference creation.

## Supplementary Material



## Notes

Remediation details including a summary of the file edits, examples of simulated spectra remediations, assessment of the similarity metric, and assessments of the improvements of CASMDB relative to the original data (PDF)

## Abbreviations

CASMDB: CcpNmr Analysis Simulated Metabolomics Database
NMR: nuclear magnetic resonance
MS: mass spectrometry
HMDB: Human Metabolome Database
BMRB: Biological Magnetic Resonance Data Bank
GISSMO: Guided Ideographic Spin System Model Optimization
SSMs: spin system matrices
1D 1H: one-dimensional proton
SQL: Structured Query Language
SMILES: Simplified Molecular-Input Line-Entry System
InChI: International Chemical Identifier number
