# Supplementary material for: CASMDB: An Open-Source Database of Metabolite Annotation Data for 1D 1H NMR-Based Metabolomics
Source: Anal Chem. 2026 Apr 21;98(17):12224–33. doi: 10.1021/acs.analchem.5c04525 (PMC13150813; doi:10.1021/acs.analchem.5c04525)
Supplement: Supplementary file 1 [file ac5c04525_si_001.pdf]

CASMDB: An Open-Source Database of Metabolite Annotation Data for 1D ^{1}H NMR-Based Metabolomics

Supplementary Data

Morgan W. Hayward[1], Luca G. Mureddu[1], Gary S. Thompson[2], Marie M. Phelan[3], Edward J. Brooksbank[1] and Geerten W. Vuister[1]**

[1] Devision of Molecular and Cell Biology, Leicester Institute of Structural and Chemical Biology, University of Leicester, Henry Wellcome Building, Lancaster Road, Leicester LE1 7HN, United Kingdom

[2] School of Natural Sciences, University of Kent, Canterbury, CT2 7NZ, United Kingdom

[3] Liverpool Shared Research Facilities (LivSRF) & Institute of Systems, Molecular and Integrative Biology, NMR Centre for Structural Biology, University of Liverpool, Crown Street, Liverpool, L69 7ZB, United Kingdom

* Corresponding authors: gv29@leicester.ac.uk or mphelan@liverpool.ac.uk

Supplementary Data – Table of Contents

**Supplementary Table 1.**	**S3**
**Supplementary Figure 1.**	**S4**
**Supplementary Figure 2.**	**S5**
**Supplementary Figure 3.**	**S6**
**Supplementary Figure 4.**	**S7**
**Supplementary Table 2.**	**S8**
**Supplementary Table 3.**	**S9**
**Supplementary Table 4.**	**S10-S11**
**Supplementary Figure 5.**	**S12**
**Supplementary Figure 6.**	**S13**
**Supplementary Table 5a.**	**S14-S15**
**Supplementary Table 5b.**	**S16-S17**
**Supplementary Table 5c.**	**S18-S19**

Supplementary Table 1. A record of remediation details of metabolite reference files from the BMRB and HMDB.

Data Source	File Count	File Type	Refinement or Exclusion	Details
HMDB	208	nmrML	Refinement	Adding ‘/’ to the closing statement on line 5 and replacing ‘</chemicalShiftStandard>’ with ‘/>’ at the closing statement on line 11
	4	nmrML	Refinement	Removing the ‘>’ character from the opening statement
	3	nmrML	Refinement	Removing the ‘<’ from the value string
	1	nmrML	Refinement	Replaced ‘-‘ with ‘Nan’ to allow conversion into float
	5	nmrML	Exclusion	No values for peaks or multiplets
	12	TXT	Refinement	Added ‘\t’ to allow division of data
	11	TXT	Refinement	Added ‘-‘ to empty space to recognise Null values
	16	TXT	Refinement	Swapped assignment and multiplet table titles for correct recognition
	1	TXT	Refinement	Removed a rogue character (‘v’) from file
	2	TXT	Refinement	Added a missing column title
	1	TXT	Refinement	Added a new line character after a table title
	1	TXT	Refinement	Removed an empty column with no title
BMRB	1	STAR	Refinement	Edited filename for real spectrum.
	600	STAR	Exclusion	No spectral peak list or assigned chemical shift
	46	STAR	Exclusion	Unsuitable peak chemical shift data
	768	STAR	Exclusion	Unsuitable peak intensity data
GISSMO	6	XML	Refinement	Spin system label changed to ‘merged’.

STAR, TXT, XML and nmrML denote Self-Defining Text Archive and Retrieval, text format, Extensible Markup Language, and NMR Markup Language format, respectively.

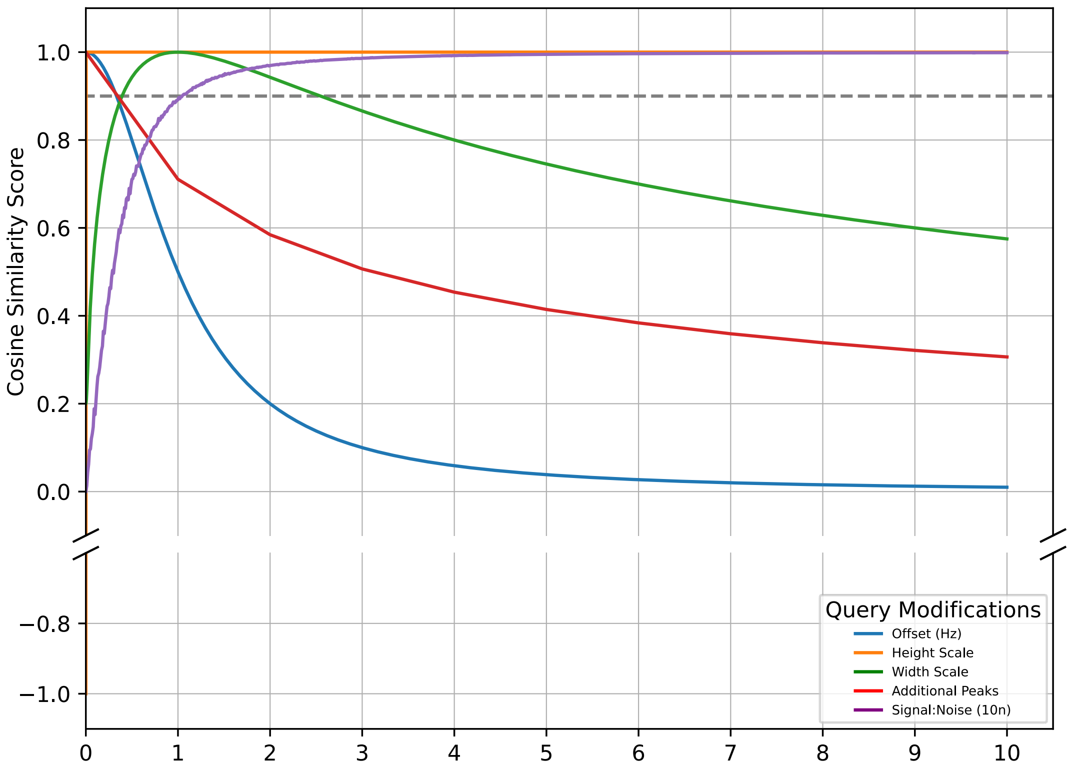

Supplementary Figure 1. Assessment of sensitivities of the cosine similarity metric (eq. 1 main document; vertical) on various factors. The scale of the horizontal axis is variable dependent the factor examined: for offset (blue) Hz; height (orange) scale factor; line width (green) width scale factor, additional unrelated peaks (red) count; signal-to-noise (purple) SNR x10. Cosine similarity is sensitive to offset and interference of other spectrum features, yet suitably resistant to peak width differences, insensitive to scale and sensitive only to moderate noise. The grey dashed line shows the acceptance threshold used to accept/reject simulated spectra.

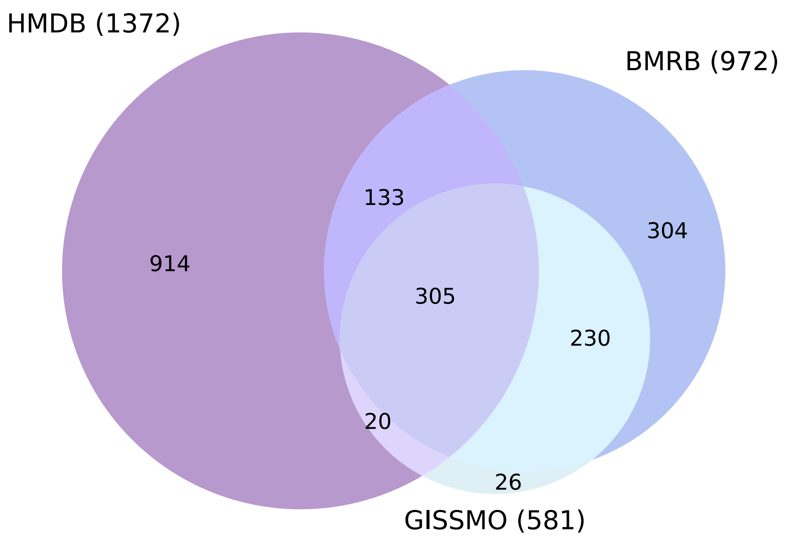

Supplementary Figure 2. Venn diagram showing CASMDB metabolite distribution as derived from the source databases. Numbers indicate unique metabolite counts. Metabolites were identified as identical across databases on the basis of their InChI codes.

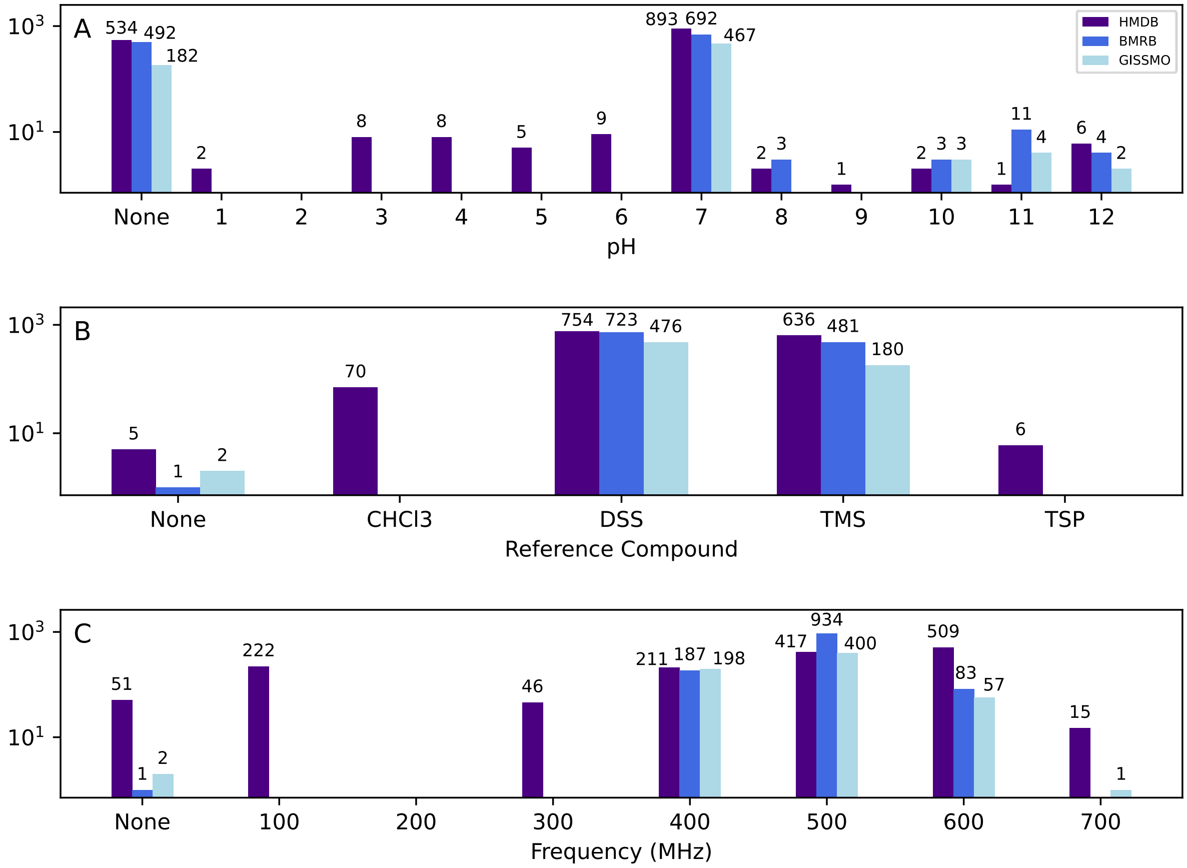

Supplementary Figure 3. Experimental conditions of the CASMDB entries colour coded according to source. Count numbers are shown above the bars, and all vertical axes are in logarithmic scale. A) Bar chart depicting the distribution of pH values in the samples, rounded to nearest integer values. B) Bar chart depicting the spectral referencing compound used. C) Bar chart depicting the distribution of spectrometer frequencies in the metabolite spectra, rounded to the nearest 100 MHz. The None value indicates the entries with an unspecified value.

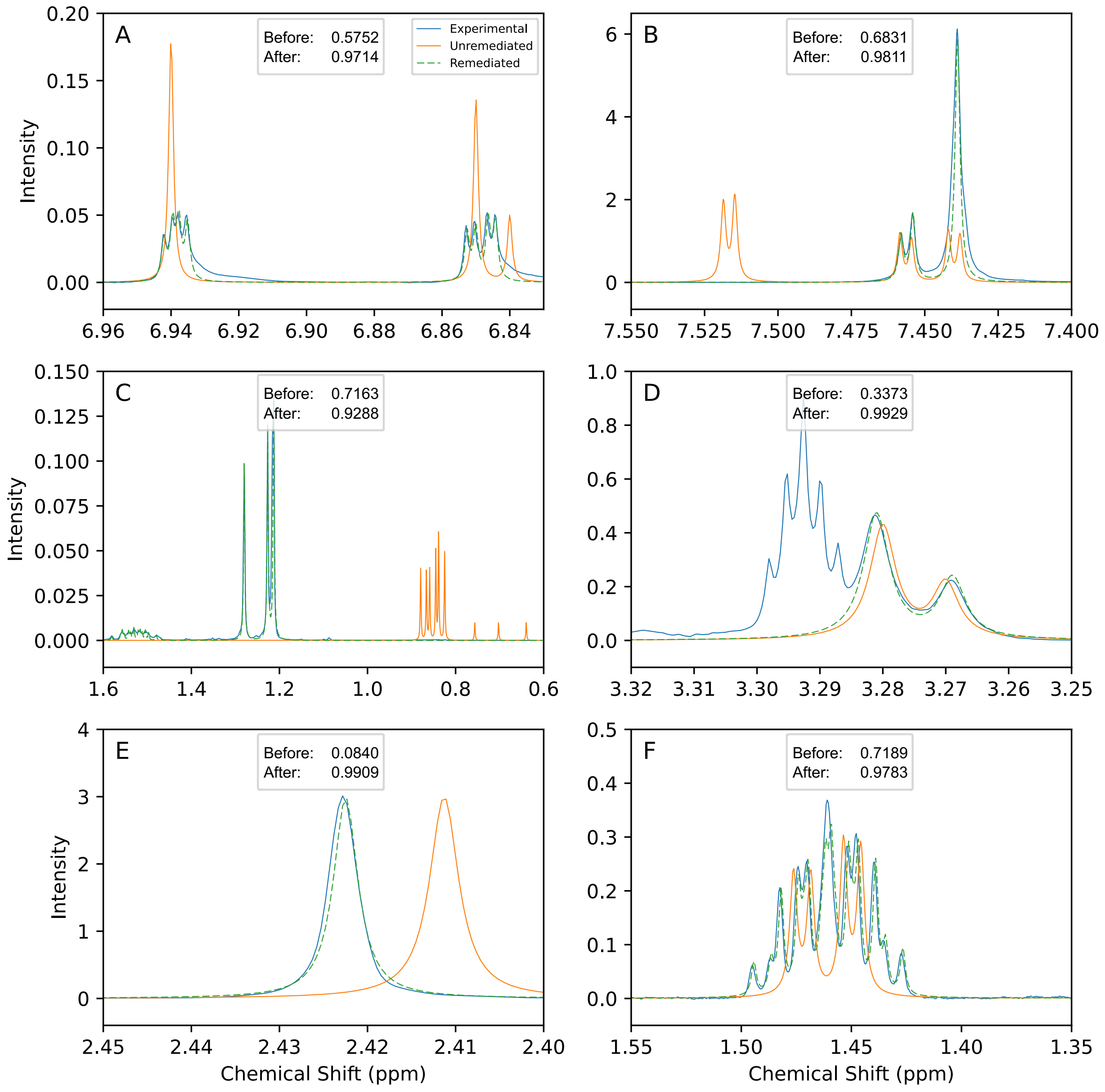

Supplementary Figure 4. Remediation examples. All spectra are colour-coded to represent the experimental spectra (blue), original simulation before remediation (orange) and the accepted simulation after remediation (green dashed). Cosine similarity scores for the visible regions, before and after remediation are shown included in each panel. A) Low peak precision in peak annotations. Peaks with chemical shift recorded to a precision of +-0.005ppm could not be used to recreate accurate spectral line-shapes. B) Discrepancies between peak lists and the experimental spectra as well as additional peaks. C) Extreme differences between experimental and simulated spectra, possibly due to peak-picking of a low-intensity region instead of the regions of high signal. D) interference in the metabolite signal by the solvent signal. In this example, the Deuterated methanol quintuplet is close enough to the metabolite signal to affect the similarity. scoring. E) Chemical shift differences in the peak locations. F) Missing couplings from a spin-system styled simulation.

Supplementary Table 2. Experimental standard mixture containing 22 typical metabolites prepared in 99.9% deuterated water with 100 mM sodium phosphate pH 7.6, pH 7.4 and pH 7.2.

**Metabolite ID**	**Metabolite Name**	**Concentration (**uM)
n/a	Trimethylsilyl propionate	100
HMDB0000094	Citric-acid	100
HMDB0000064	Creatine	100
HMDB0000562	Creatinine	100
HMDB0000122	D-Glucose	100
HMDB0000123	Glycine	100
HMDB0000161	L-Alanine	100
HMDB0000191	L-Aspartic acid	25
HMDB0000517	L-Arginine	100
HMDB0000168	L-Asparagine	100
HMDB0000148	L-Glutamic acid	50
HMDB0000641	L-Glutamine	100
HMDB0000177	L-Histidine	100
HMDB0000172	L-Isoleucine	100
HMDB0000687	L-Leucine	100
HMDB0000182	L-Lysine	100
MHDB0000696	L-Methionine	100
HMDB0000159	L-Phenylalanine	100
HMDB0000162	L-Proline	100
HMDB0003406	L-Serine	100
HMDB0000167	L-Threonine	100
HMDB0000158	L-Tyrosine	100
HMDB0000883	L-Valine	100

Supplementary Table 3. Spectra acquisition parameters for experimental reference mixture.

General Parameters	Value
Pulse program	cpmgpr1d
CPMG total echo time	80 ms
CPMG echos	128
Scans:	128
Spectral Width:	17.2 ppm
Number of Points:	64K
Acquisition Time:	3 seconds
Interscan delay	4 seconds

Supplementary Table 4. Cosine improvement scores after remediation for experimental standard mixture at 600MHz, cosine score color scale presented as percentile change 0% to 50% (red to yellow) and 50% to 100% (yellow to green), remediation change 3-color scale from -1 to 0 (blue to white) and 0 to +1 (white to red).

			original database scores			CASMDB remediated			multiplet	remediation change		
Name	low_ bound	high_ bound	HMDB	BMRB	GISSMO	HMDB	BMRB	GISSMO	alignment	HMDB	BMRB	GISSMO
Citrate	2.52	2.56			0.99			0.99	yes			0.00
Citrate	2.64	2.68			0.85			0.88				0.03
Creatine	3.04	3.04		0.99	1.00		1.00	0.99	yes		0.01	0.00
Creatine	3.93	3.94		0.16	0.21		0.19	0.99			0.04	0.78
Creatinine	3.04	3.05			0.99			0.99	yes			0.00
Creatinine	4.05	4.06			0.70			0.60				-0.09
D-Glucose	3.23	3.27		0.74	0.76		0.71	0.75	yes		-0.02	-0.01
D-Glucose	3.39	3.41		0.81	0.86		0.83	0.87			0.02	0.01
D-Glucose	3.41	3.44		0.89	0.92		0.85	0.93			-0.04	0.02
D-Glucose	3.45	3.49		0.90	0.86		0.89	0.98			-0.02	0.12
D-Glucose	3.48	3.52		0.97	0.91		0.96	0.98			-0.01	0.06
D-Glucose	3.52	3.55		0.82	0.99		0.81	0.98			-0.01	-0.01
D-Glucose	3.70	3.72		0.88	0.84		0.85	0.97			-0.03	0.13
D-Glucose	3.72	3.75		0.89	0.95		0.86	0.96			-0.03	0.01
D-Glucose	3.75	3.79		0.52	0.52		0.47	0.78			-0.05	0.26
D-Glucose	3.82	3.87		0.62	0.63		0.55	0.76			-0.08	0.13
D-Glucose	3.88	3.92		0.67	0.71		0.65	0.88			-0.02	0.17
D-Glucose	4.64	4.66		0.22	0.43		0.22	0.96			0.00	0.53
D-Glucose	5.23	5.25		0.73	0.89		0.68	0.99			-0.05	0.10
Glycine	3.56	3.57		0.99	0.97		0.98	0.98	yes		-0.02	0.01
L-Alanine	1.47	1.49			0.98			0.99	yes			0.01
L-Alanine	3.76	3.81			0.49			0.48				-0.01
L-Arginine	1.62	1.70		0.80	0.68		0.70	0.83	yes		-0.10	0.15
L-Arginine	1.70	1.78		0.86	0.78		0.77	0.91			-0.10	0.14
L-Arginine	1.86	1.96		0.42	0.33		0.39	0.66			-0.04	0.33
L-Arginine	3.23	3.27		0.52	0.48		0.43	0.94			-0.09	0.47
L-Arginine	3.75	3.79		0.56	0.55		0.56	0.86			0.00	0.31
L-Asparagine	2.83	2.89		0.95	0.94		0.95	0.95	yes		0.00	0.01
L-Asparagine	2.93	2.97		0.38	0.39		0.39	0.97			0.01	0.58
L-Asparagine	3.98	4.02		0.79	0.83		0.83	0.83			0.04	0.01
L-Aspartic Acid	2.66	2.71		0.76	0.78		0.74	0.77			-0.02	-0.02
L-Aspartic Acid	2.80	2.84		0.55	0.54		0.59	0.90			0.03	0.36
L-Aspartic Acid	3.88	3.92		0.69	0.66		0.68	0.92			-0.01	0.25
l-glutamic_acid	2.02	2.10		0.85	0.81		0.81	0.85	yes		-0.05	0.03
l-glutamic_acid	2.10	2.17		0.49	0.39		0.48	0.62			-0.01	0.22
l-glutamic_acid	2.31	2.40		0.78	0.73		0.75	0.98			-0.03	0.25
l-glutamic_acid	3.75	3.78		0.87	0.88		0.88	0.89			0.02	0.01
l-glutamine	2.10	2.18			0.62			0.59	yes			-0.04
l-glutamine	2.41	2.51			0.61			0.95				0.34
l-glutamine	3.75	3.79			0.57			0.94				0.37
l-histidine	3.11	3.17	0.81	0.74	0.72	0.73	0.72	0.73	yes	-0.08	-0.02	0.01
l-histidine	3.22	3.27	0.51	0.35	0.37	0.62	0.35	0.62		0.12	0.00	0.25
l-histidine	3.97	4.02	0.47	0.29	0.57	0.81	0.58	0.80		0.34	0.29	0.23
l-histidine	7.06	7.08	0.08	0.26	0.44	0.93	0.44	0.95		0.86	0.18	0.50
l-histidine	7.81	7.83	0.38	0.20	0.64	0.95	0.76	0.96		0.57	0.56	0.32
l-isoleucine	0.93	0.96	0.91	0.92	0.91	0.91	0.90	0.92	yes	0.00	-0.02	0.01
l-isoleucine	1.01	1.02	0.97	0.98	1.00	0.99	0.96	1.00		0.03	-0.02	0.00
l-isoleucine	1.23	1.31	0.82	0.87	0.81	0.85	0.84	0.92		0.03	-0.03	0.11
l-isoleucine	1.44	1.52	0.55	0.52	0.44	0.67	0.42	0.72		0.13	-0.10	0.28
l-isoleucine	1.95	2.02	0.82	0.81	0.82	0.77	0.76	0.81		-0.05	-0.05	-0.01
l-isoleucine	3.67	3.69	0.29	0.92	0.75	0.98	0.94	0.99		0.69	0.02	0.24
l-leucine	0.95	0.98		0.96	0.94		0.94	0.97	yes		-0.02	0.04
l-leucine	1.65	1.79		0.88	0.76		0.75	0.83			-0.13	0.07
l-leucine	3.72	3.75		0.89	0.83		0.80	0.78			-0.09	-0.05
l-lysine	1.41	1.56		0.45	0.43		0.34	0.47	yes		-0.11	0.04

l-lysine	1.70	1.78		0.78	0.67		0.65	0.93			-0.14	0.27
l-lysine	1.85	1.96		0.47	0.55		0.46	0.48			-0.01	-0.07
l-lysine	3.01	3.04		0.20	0.19		0.17	0.57			-0.03	0.38
l-lysine	3.75	3.78		0.62	0.53		0.58	0.76			-0.04	0.23
l-methionine	2.10	2.17		0.96	0.97		0.96	0.97	yes		0.00	0.01
l-methionine	2.17	2.24		0.75	0.67		0.73	0.96			-0.01	0.29
l-methionine	2.63	2.67		0.93	0.91		0.89	0.86			-0.04	-0.05
l-methionine	3.85	3.88		0.41	0.53		0.39	0.90			-0.03	0.36
l-phenylalanine	3.11	3.16		0.90	0.86		0.86	0.86	yes		-0.04	0.00
l-phenylalanine	3.27	3.31		0.99	0.96		0.98	0.97			-0.02	0.01
l-phenylalanine	7.32	7.35		0.92	0.84		0.90	0.98			-0.02	0.14
l-phenylalanine	7.36	7.40		0.99	0.95		0.95	0.96			-0.04	0.01
l-phenylalanine	7.41	7.45		0.98	0.96		0.93	0.99			-0.05	0.03
l-proline	1.96	2.10	0.83	0.86	0.85	0.77	0.87	0.88	yes	-0.06	0.01	0.03
l-proline	1.96	2.10	0.83	0.86	0.85	0.77	0.80	0.88		-0.06	-0.06	0.03
l-proline	2.32	2.39	0.86	0.86	0.84	0.80	0.85	0.86		-0.05	-0.01	0.03
l-proline	3.32	3.37	0.78	0.75	0.79	0.95	0.70	0.98		0.18	-0.04	0.19
l-proline	3.40	3.45	0.56	0.73	0.76	0.82	0.69	0.83		0.26	-0.05	0.08
l-proline	4.12	4.15	0.85	0.85	0.84	0.96	0.91	0.97		0.11	0.06	0.12
l-serine	3.83	3.85		0.94	0.95		0.94	0.96	yes		-0.01	0.01
l-serine	3.93	3.97		0.49	0.49		0.46	0.54			-0.03	0.05
l-serine	3.94	3.97		0.95	0.98		0.89	0.99			-0.06	0.01
l-serine	3.98	4.01		0.65	0.65		0.65	0.78			0.00	0.13
l-threonine	1.32	1.35		0.99	0.99		0.99	0.99	yes		0.00	0.00
l-threonine	3.57	3.59		0.31	0.29		0.34	0.97			0.03	0.68
l-threonine	4.23	4.28		0.50	0.51		0.47	0.91			-0.03	0.40
l-valine	0.98	1.00		0.94	1.00		0.99	0.99	yes		0.05	-0.01
l-valine	1.03	1.06		0.70	0.53		0.67	0.99			-0.03	0.46
l-valine	2.24	2.32		0.85	0.74		0.81	0.95			-0.04	0.21
l-valine	3.60	3.63		0.64	0.49		0.61	0.98			-0.04	0.49

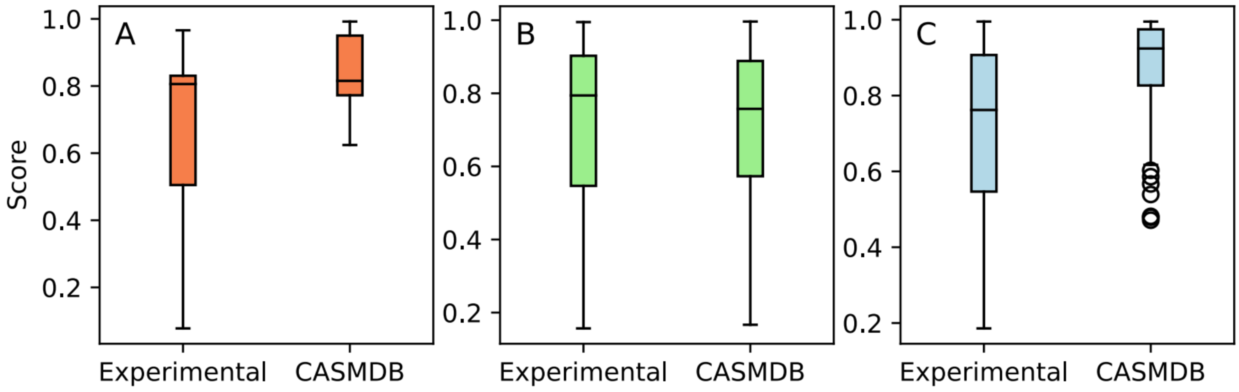

Supplementary Figure 5. Box plots of the similarity scores (eq. 1) of the original spectra (HMDB, BMRB and GISSMO respectively) vs the CASMDB simulated spectra when validated against the 600 MHz, pH 7.4 experimental mixture spectrum.

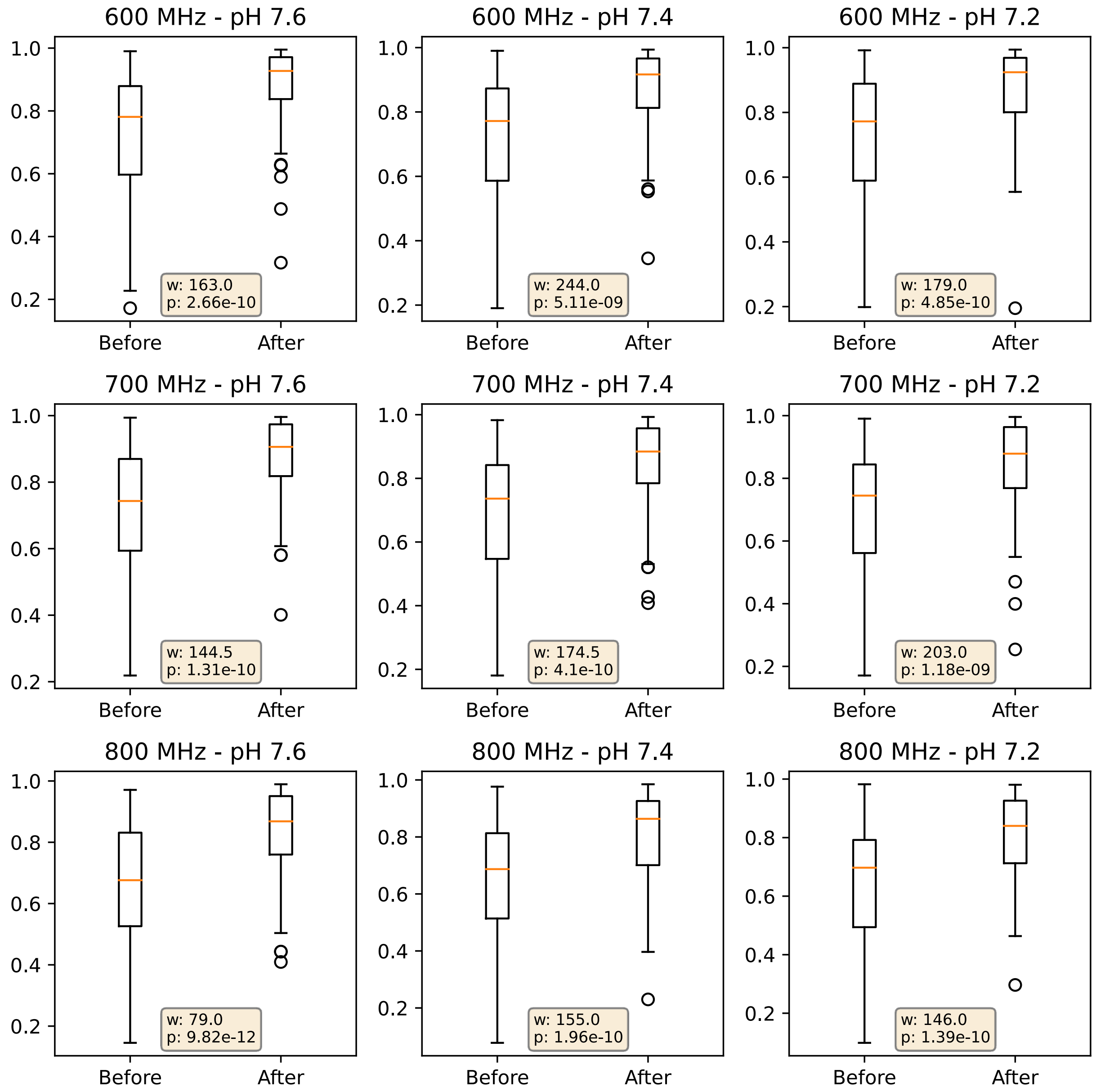

Supplementary Figure 6. Match score summary of the validation of the CASMDB entries against the equivalent static spectra from the GISSMO library, for three pH values (7.6, 7.4, 7.2) at three field strengths (600, 700, 800 MHz). In all examples the CASMDB applications show a convergence of scores towards a similarity score of 1.0. w-values and p-values are determined by Wilcoxon pairwise non-parametric test.

Supplementary Table 5a Cosine improvement scores for GISSMO after remediation for experimental standard mixture at 600 MHz pH 7.2, 7.4 and 7.6, remediation change 3-color scale from -1 to 0 (blue to white) and 0 to +1 (white to red). Metabolite name (name) minimum bound (min), maximal bound (max) Original score (orig), CASMDB score (CASM), difference in score (diff) region integration original (o-int) region integration CASMDB (c-int).

name	min	max	orig	CASM	diff	o-int	c-int	orig	CASM	diff	o-int	c-int	orig	CASM	diff	o-int	c-int
			pH7.2					pH 7.4					pH 7.6				
citric acid	2.5200	2.5600	0.995	0.984	-0.012	1067.04	942.92	0.995	0.869	-0.127	963.77	942.17	0.992	0.002	-0.990	1023.19	0.03
citric acid	2.6400	2.6800	0.515	0.930	0.415	1953.61	943.80	0.712	0.883	0.171	1856.96	943.75	0.866	0.321	-0.544	1882.25	1926.99
creatine	3.0353	3.0436	0.985	0.986	0.001	1545.72	1267.09	0.990	0.987	-0.003	1525.71	1263.25	0.990	0.988	-0.003	1541.82	1263.44
creatine	3.9278	3.9373	0.199	0.987	0.789	994.12	863.90	0.190	0.991	0.801	983.68	861.80	0.172	0.990	0.818	990.86	861.22
creatinine	3.0430	3.0510	0.992	0.992	0.000	1437.32	1250.08	0.991	0.994	0.003	1419.37	1253.54	0.991	0.992	0.000	1411.78	1245.69
creatinine	4.0500	4.0600	0.735	0.776	0.041	78.76	848.01	0.702	0.756	0.053	73.13	843.05	0.697	0.751	0.054	74.96	831.98
alpha-d-glucose	4.6395	4.6621	0.393	0.974	0.581	252.20	453.55	0.432	0.976	0.544	261.01	453.66	0.375	0.974	0.599	250.68	453.26
alpha-d-glucose	3.8822	3.9161	0.703	0.882	0.180	732.65	471.81	0.710	0.871	0.161	725.60	471.67	0.700	0.872	0.172	744.49	474.30
alpha-d-glucose	3.7247	3.7456	0.934	0.901	-0.034	540.76	385.26	0.953	0.915	-0.038	538.79	385.85	0.959	0.966	0.007	559.04	382.93
alpha-d-glucose	3.4519	3.4890	0.918	0.984	0.067	445.88	640.53	0.900	0.984	0.084	432.32	639.51	0.892	0.986	0.094	438.62	639.81
alpha-d-glucose	3.3871	3.4104	0.898	0.867	-0.031	471.71	404.55	0.920	0.884	-0.037	505.64	404.83	0.873	0.876	0.003	521.71	401.78
alpha-d-glucose	3.4764	3.5153	0.975	0.988	0.013	400.15	586.96	0.951	0.983	0.032	392.29	588.17	0.951	0.985	0.034	403.98	589.79
alpha-d-glucose	3.2300	3.2681	0.784	0.798	0.013	1595.43	467.93	0.772	0.774	0.002	1585.10	468.06	0.751	0.752	0.002	1529.41	467.03
alpha-d-glucose	3.7464	3.7875	0.761	0.693	-0.068	2056.24	490.82	0.757	0.685	-0.072	2105.70	491.55	0.521	0.742	0.221	2144.12	492.87
alpha-d-glucose	3.8194	3.8666	0.751	0.756	0.006	1234.77	939.01	0.731	0.703	-0.028	1276.46	954.42	0.657	0.813	0.156	1327.51	844.26
alpha-d-glucose	3.8194	3.8666	0.751	0.756	0.006	1234.77	939.01	0.731	0.703	-0.028	1276.46	954.42	0.657	0.813	0.156	1327.51	844.26
alpha-d-glucose	3.4109	3.4379	0.938	0.925	-0.013	586.47	550.58	0.951	0.930	-0.021	561.06	552.15	0.944	0.905	-0.039	550.22	555.08
alpha-d-glucose	3.6998	3.7246	0.860	0.935	0.075	323.17	567.25	0.867	0.944	0.077	314.59	564.82	0.873	0.951	0.078	329.79	562.45
alpha-d-glucose	3.5219	3.5547	0.963	0.988	0.024	236.01	473.03	0.977	0.976	-0.001	240.77	472.72	0.971	0.986	0.014	239.70	472.82
alpha-d-glucose	5.2301	5.2452	0.842	0.985	0.143	201.85	446.65	0.882	0.985	0.103	196.87	446.66	0.869	0.989	0.120	195.66	446.61
glycine	3.5558	3.5658	0.989	0.987	-0.002	984.85	865.80	0.990	0.985	-0.005	1005.24	867.12	0.987	0.990	0.003	1032.60	869.71
l-alanine	1.4736	1.4943	0.941	0.988	0.047	1869.72	1352.12	0.936	0.987	0.051	1861.24	1354.32	0.935	0.991	0.056	1875.61	1354.25
l-alanine	3.7645	3.8099	0.732	0.579	-0.153	1404.19	475.54	0.671	0.524	-0.147	1400.35	475.05	0.585	0.458	-0.127	1308.80	473.02
l-arginine	1.8586	1.9571	0.452	0.661	0.209	4232.25	979.94	0.451	0.659	0.208	4085.36	979.95	0.430	0.630	0.200	4288.28	979.05
l-arginine	1.8586	1.9571	0.452	0.661	0.209	4232.25	979.94	0.451	0.659	0.208	4085.36	979.95	0.430	0.630	0.200	4288.28	979.05
l-arginine	1.7030	1.7788	0.794	0.787	-0.007	2642.07	486.34	0.801	0.795	-0.006	2612.05	486.35	0.809	0.812	0.003	2609.63	486.59
l-arginine	1.6174	1.7023	0.725	0.698	-0.028	994.68	492.54	0.730	0.703	-0.028	991.06	492.48	0.723	0.698	-0.026	984.21	492.61
l-arginine	3.7542	3.7870	0.524	0.748	0.224	1712.12	932.74	0.514	0.779	0.264	1764.41	936.52	0.591	0.842	0.251	1810.74	943.98
l-arginine	3.2290	3.2678	0.509	0.922	0.414	1596.10	477.25	0.481	0.917	0.437	1585.78	477.23	0.528	0.911	0.383	1537.52	477.02
l-asparagine	2.8327	2.8856	0.932	0.930	-0.002	609.92	452.51	0.931	0.930	-0.001	622.13	468.37	0.945	0.947	0.002	621.99	476.99
l-asparagine	2.9256	2.9739	0.422	0.982	0.561	507.64	424.76	0.398	0.986	0.588	532.22	454.30	0.407	0.973	0.566	572.31	476.63
l-asparagine	3.9829	4.0170	0.643	0.772	0.129	1873.20	431.46	0.604	0.799	0.196	1889.11	465.42	0.835	0.806	-0.029	1810.83	474.09
l-glutamic acid	2.3104	2.4005	0.772	0.975	0.203	1658.72	977.80	0.768	0.972	0.204	1603.74	978.05	0.762	0.976	0.214	1628.66	977.73
l-glutamic acid	2.3104	2.4005	0.772	0.975	0.203	1658.72	977.80	0.768	0.972	0.204	1603.74	978.05	0.762	0.976	0.214	1628.66	977.73
l-glutamic acid	2.1021	2.1686	0.499	0.566	0.067	3508.94	485.09	0.477	0.584	0.107	3539.63	485.22	0.477	0.631	0.154	3443.36	485.48
l-glutamic acid	2.0234	2.0974	0.861	0.833	-0.029	1396.71	488.54	0.856	0.825	-0.031	1395.77	488.69	0.861	0.832	-0.029	1394.84	488.98
l-glutamic acid	3.7464	3.7764	0.841	0.835	-0.007	1568.36	468.31	0.826	0.825	-0.002	1639.69	467.82	0.896	0.874	-0.022	1745.95	466.53
l-glutamine	2.4086	2.5052	0.915	0.959	0.045	1105.71	977.52	0.828	0.961	0.133	1080.81	977.52	0.905	0.958	0.053	1058.15	977.58
l-glutamine	2.4086	2.5052	0.915	0.959	0.045	1105.71	977.52	0.828	0.961	0.133	1080.81	977.52	0.905	0.958	0.053	1058.15	977.58
l-glutamine	2.1002	2.1796	0.639	0.586	-0.053	3633.14	953.98	0.578	0.498	-0.080	3661.85	962.85	0.578	0.470	-0.107	3572.27	966.66
l-glutamine	2.1002	2.1796	0.639	0.586	-0.053	3633.14	953.98	0.578	0.498	-0.080	3661.85	962.85	0.578	0.470	-0.107	3572.27	966.66
l-glutamine	3.7549	3.7881	0.775	0.855	0.080	1739.31	467.15	0.864	0.830	-0.034	1786.52	464.05	0.630	0.934	0.304	1811.22	471.97
l-histidine	3.1082	3.1650	0.574	0.549	-0.025	780.59	3.26	0.703	0.680	-0.023	879.57	3.84	0.507	0.484	-0.024	1027.56	7.36
l-histidine	3.2197	3.2679	0.228	0.184	-0.044	1663.49	46.44	0.274	0.342	0.069	1627.08	28.05	0.351	0.307	-0.044	1697.23	27.10
l-histidine	7.0626	7.0834	0.624	0.914	0.290	430.41	466.10	0.646	0.904	0.258	483.29	466.10	0.631	0.905	0.274	488.71	467.27
l-histidine	7.8054	7.8263	0.762	0.749	-0.012	11.01	0.77	0.761	0.732	-0.028	9.81	2.08	0.643	0.922	0.279	413.39	467.43
l-histidine	3.9715	4.0156	0.578	0.839	0.261	2066.11	478.45	0.578	0.811	0.233	2186.86	479.47	0.627	0.792	0.165	2209.83	480.94
l-isoleucine	0.9263	0.9610	0.915	0.907	-0.008	2697.00	1413.06	0.917	0.911	-0.006	2673.94	1413.10	0.915	0.908	-0.008	2657.50	1413.97
l-isoleucine	1.4397	1.5166	0.578	0.721	0.143	2837.00	485.48	0.575	0.719	0.144	2820.87	485.51	0.577	0.716	0.139	2833.95	485.58
l-isoleucine	1.2251	1.3119	0.907	0.925	0.018	1044.32	487.03	0.900	0.922	0.022	1026.77	486.96	0.898	0.921	0.023	1081.11	486.92
l-isoleucine	1.9464	2.0248	0.815	0.796	-0.019	1412.97	487.33	0.819	0.800	-0.019	1398.30	487.31	0.821	0.802	-0.019	1405.24	487.31
l-isoleucine	1.0055	1.0247	0.992	0.988	-0.004	1675.78	1336.60	0.990	0.992	0.002	1649.01	1336.50	0.990	0.992	0.002	1651.48	1333.11
l-isoleucine	3.6668	3.6865	0.838	0.991	0.153	528.42	460.01	0.884	0.994	0.110	528.23	459.50	0.985	0.990	0.004	541.48	455.44
l-leucine	0.9494	0.9815	0.961	0.973	0.012	3684.17	2789.63	0.961	0.973	0.012	3640.32	2789.74	0.961	0.972	0.011	3628.78	2790.10
l-leucine	1.6509	1.7854	0.847	0.826	-0.021	3500.66	1464.20	0.846	0.824	-0.022	3461.72	1464.20	0.848	0.823	-0.026	3454.43	1464.27
l-leucine	0.9494	0.9815	0.961	0.973	0.012	3684.17	2789.63	0.961	0.973	0.012	3640.32	2789.74	0.961	0.972	0.011	3628.78	2790.10
l-leucine	1.6509	1.7854	0.847	0.826	-0.021	3500.66	1464.20	0.846	0.824	-0.022	3461.72	1464.20	0.848	0.823	-0.026	3454.43	1464.27
l-leucine	1.6509	1.7854	0.847	0.826	-0.021	3500.66	1464.20	0.846	0.824	-0.022	3461.72	1464.20	0.848	0.823	-0.026	3454.43	1464.27
l-leucine	3.7249	3.7548	0.865	0.858	-0.008	885.45	434.88	0.875	0.879	0.004	881.64	436.83	0.873	0.917	0.044	911.19	419.74

l-lysine	3.7452	3.7763	0.557	0.709	0.152	1573.73	329.62	0.552	0.736	0.184	1656.43	337.29	0.619	0.821	0.202	1764.76	355.58
l-lysine	1.8529	1.9568	0.524	0.640	0.116	4241.70	925.22	0.519	0.596	0.077	4091.33	925.81	0.530	0.664	0.135	4292.50	926.69
l-lysine	1.8529	1.9568	0.524	0.640	0.116	4241.70	925.22	0.519	0.596	0.077	4091.33	925.81	0.530	0.664	0.135	4292.50	926.69
l-lysine	1.4096	1.5606	0.461	0.494	0.034	3290.61	979.02	0.459	0.491	0.032	3263.23	979.02	0.458	0.470	0.012	3277.01	978.57
l-lysine	1.4096	1.5606	0.461	0.494	0.034	3290.61	979.02	0.459	0.491	0.032	3263.23	979.02	0.458	0.470	0.012	3277.01	978.57
l-lysine	1.7012	1.7802	0.761	0.947	0.186	2672.72	962.20	0.772	0.946	0.174	2642.60	962.18	0.800	0.947	0.147	2640.75	961.75
l-lysine	1.7012	1.7802	0.761	0.947	0.186	2672.72	962.20	0.772	0.946	0.174	2642.60	962.18	0.800	0.947	0.147	2640.75	961.75
l-lysine	3.0111	3.0428	0.229	0.531	0.302	2254.87	903.35	0.224	0.530	0.306	2242.77	906.78	0.227	0.573	0.345	2262.17	916.07
l-methionine	2.1015	2.1677	0.969	0.967	-0.002	3503.73	1872.78	0.964	0.970	0.006	3537.30	1872.65	0.961	0.964	0.003	3441.13	1875.87
l-methionine	2.1693	2.2390	0.901	0.934	0.034	739.30	507.54	0.862	0.944	0.083	733.41	513.91	0.803	0.937	0.134	731.94	516.24
l-methionine	2.1015	2.1677	0.969	0.967	-0.002	3503.73	1872.78	0.964	0.970	0.006	3537.30	1872.65	0.961	0.964	0.003	3441.13	1875.87
l-methionine	3.8339	3.8539	0.895	0.840	-0.054	683.63	437.88	0.929	0.884	-0.045	762.86	498.46	0.725	0.872	0.147	820.60	558.07
l-methionine	3.8474	3.8774	0.586	0.921	0.335	857.44	646.17	0.542	0.916	0.374	830.70	605.32	0.552	0.930	0.377	798.92	569.92
l-methionine	2.6296	2.6653	0.939	0.927	-0.012	1694.72	470.23	0.963	0.940	-0.023	1649.54	470.60	0.942	0.889	-0.053	1628.97	470.46
l-phenylalanine	7.3594	7.3988	0.951	0.952	0.001	560.19	489.36	0.965	0.952	-0.013	565.08	489.75	0.977	0.946	-0.031	579.09	490.48
l-phenylalanine	7.4115	7.4508	0.925	0.994	0.069	1073.02	954.39	0.947	0.994	0.047	1063.04	954.39	0.974	0.993	0.019	1080.96	954.17
l-phenylalanine	7.3180	7.3492	0.991	0.964	-0.027	1075.03	947.44	0.989	0.964	-0.025	1094.53	947.79	0.931	0.964	0.032	1098.46	947.72
l-phenylalanine	3.1100	3.1564	0.953	0.952	-0.001	723.57	461.94	0.957	0.909	-0.049	728.07	465.32	0.915	0.841	-0.074	876.03	469.75
l-phenylalanine	3.2679	3.3097	0.912	0.934	0.022	630.47	468.51	0.933	0.910	-0.024	585.21	470.46	0.969	0.959	-0.010	543.51	469.09
l-phenylalanine	3.9859	4.0059	0.855	0.914	0.059	1249.24	345.32	0.860	0.933	0.073	1320.46	342.47	0.875	0.758	-0.117	1226.60	293.29
l-proline	1.9568	2.1007	0.881	0.872	-0.009	2706.09	1444.86	0.882	0.872	-0.010	2691.11	1444.77	0.870	0.868	-0.002	2691.15	1443.93
l-proline	1.9568	2.1007	0.881	0.872	-0.009	2706.09	1444.86	0.882	0.872	-0.010	2691.11	1444.77	0.870	0.868	-0.002	2691.15	1443.93
l-proline	1.9568	2.1007	0.881	0.872	-0.009	2706.09	1444.86	0.882	0.872	-0.010	2691.11	1444.77	0.870	0.868	-0.002	2691.15	1443.93
l-proline	2.3213	2.3880	0.853	0.838	-0.014	1525.62	477.96	0.853	0.837	-0.016	1489.93	478.11	0.854	0.833	-0.021	1485.47	477.85
l-proline	4.1168	4.1531	0.582	0.749	0.168	534.45	470.29	0.678	0.713	0.035	491.18	469.62	0.880	0.963	0.083	264.72	469.82
l-proline	3.3161	3.3665	0.875	0.982	0.107	415.79	474.57	0.786	0.983	0.197	417.98	474.46	0.727	0.979	0.253	428.92	474.29
l-proline	3.3969	3.4493	0.688	0.791	0.103	1049.29	478.85	0.681	0.777	0.096	1055.13	478.89	0.736	0.822	0.085	962.62	478.87
l-serine	3.9777	4.0140	0.600	0.725	0.125	2020.90	470.26	0.506	0.800	0.294	2087.21	469.61	0.678	0.746	0.069	2068.63	463.98
l-serine	3.9387	3.9677	0.628	0.982	0.355	441.22	396.18	0.764	0.983	0.219	439.69	396.79	0.965	0.986	0.021	465.02	398.26
l-serine	3.8295	3.8543	0.909	0.903	-0.005	795.42	395.20	0.922	0.925	0.003	884.43	436.09	0.953	0.959	0.005	947.97	462.13
l-threonine	1.3200	1.3463	0.994	0.989	-0.005	1698.40	1405.68	0.994	0.992	-0.002	1656.45	1405.78	0.995	0.989	-0.006	1683.89	1405.55
l-threonine	4.2264	4.2755	0.641	0.844	0.203	1134.50	466.89	0.581	0.865	0.284	1155.85	476.15	0.501	0.890	0.389	1193.11	479.76
l-threonine	3.5747	3.5931	0.893	0.982	0.089	320.71	317.65	0.969	0.991	0.022	448.94	405.19	0.295	0.989	0.694	521.04	454.22
l-valine	1.0299	1.0630	0.670	0.988	0.318	1671.41	1434.41	0.650	0.992	0.343	1641.24	1434.01	0.642	0.994	0.352	1661.23	1434.43
l-valine	0.9826	1.0049	0.944	0.986	0.042	1662.89	1368.29	0.939	0.989	0.051	1656.53	1368.91	0.939	0.992	0.054	1660.39	1371.79
l-valine	2.2379	2.3203	0.859	0.936	0.077	846.13	487.40	0.852	0.944	0.092	817.22	487.52	0.844	0.941	0.097	866.84	487.46
l-valine	3.5992	3.6259	0.834	0.983	0.149	453.09	471.74	0.774	0.984	0.210	447.31	471.71	0.614	0.985	0.371	468.96	472.17

Supplementary Table 5b Cosine improvement scores for GISSMO after remediation for experimental standard mixture at 700 MHz pH 7.2, 7.4 and 7.6, remediation change 3-color scale from -1 to 0 (blue to white) and 0 to +1 (white to red). Metabolite name (name) minimum bound (min), maximal bound (max) Original score (orig), CASMDB score (CASM), difference in score (diff) region integration original (o-int) region integration CASMDB (c-int).

name	min	max	orig pH 7.2	CASM	diff	o-int	c-int	orig pH 7.4	CASM	diff	o-int	c-int	orig pH 7.6	CASM	diff	o-int	c-int
citric acid	2.5200	2.5600	0.992	0.995	0.003	1744.40	950.05	0.983	0.988	0.005	1730.44	951.33	0.996	0.994	-0.002	1690.06	950.89
citric acid	2.6400	2.6800	0.372	0.834	0.462	3449.03	951.42	0.546	0.847	0.301	3424.74	951.41	0.761	0.753	-0.008	3366.99	951.34
creatine	3.0353	3.0436	0.997	0.993	-0.004	2601.52	1267.45	0.990	0.983	-0.007	2560.17	1267.40	0.994	0.991	-0.004	2559.03	1263.99
creatine	3.9278	3.9373	0.200	0.994	0.794	1572.96	861.47	0.209	0.982	0.773	1572.19	863.06	0.219	0.994	0.775	1609.24	861.28
creatinine	3.0430	3.0510	0.995	0.987	-0.008	2246.38	1255.11	0.986	0.972	-0.014	2211.56	1254.28	0.985	0.987	0.001	2222.61	1256.68
creatinine	4.0500	4.0600	0.554	0.778	0.224	178.52	444.52	0.425	0.899	0.474	201.70	559.79	0.369	0.968	0.599	384.02	746.05
alpha-d-glucose	4.6395	4.6621	0.335	0.970	0.636	450.78	459.56	0.289	0.919	0.630	353.43	459.69	0.349	0.986	0.637	497.96	459.49
alpha-d-glucose	3.8822	3.9161	0.703	0.884	0.181	1262.80	474.86	0.686	0.861	0.175	1210.59	474.88	0.695	0.905	0.209	1310.80	474.49
alpha-d-glucose	3.7247	3.7456	0.744	0.871	0.126	937.51	385.17	0.765	0.849	0.084	909.51	386.25	0.813	0.833	0.019	963.06	386.14
alpha-d-glucose	3.4519	3.4890	0.879	0.978	0.099	728.93	638.58	0.858	0.962	0.104	698.29	637.69	0.897	0.981	0.084	723.94	636.39
alpha-d-glucose	3.3871	3.4104	0.946	0.957	0.011	979.67	414.68	0.922	0.902	-0.020	899.15	413.18	0.880	0.878	-0.002	897.37	415.79
alpha-d-glucose	3.4764	3.5153	0.948	0.969	0.021	650.03	591.35	0.897	0.948	0.050	636.57	591.17	0.955	0.983	0.028	655.47	590.09
alpha-d-glucose	3.2300	3.2681	0.785	0.805	0.020	2854.36	474.37	0.731	0.749	0.018	2937.86	474.36	0.692	0.716	0.024	2762.57	473.98
alpha-d-glucose	3.7464	3.7875	0.744	0.783	0.039	3288.39	489.50	0.760	0.790	0.031	3266.23	490.62	0.576	0.845	0.269	3385.63	491.61
alpha-d-glucose	3.8194	3.8666	0.735	0.680	-0.056	2063.37	966.62	0.736	0.702	-0.034	2042.14	966.61	0.690	0.810	0.120	2189.87	966.55
alpha-d-glucose	3.8194	3.8666	0.735	0.680	-0.056	2063.37	966.62	0.736	0.702	-0.034	2042.14	966.61	0.690	0.810	0.120	2189.87	966.55
alpha-d-glucose	3.4109	3.4379	0.939	0.951	0.012	929.14	546.30	0.916	0.938	0.022	900.00	547.53	0.938	0.907	-0.031	921.29	545.22
alpha-d-glucose	3.6998	3.7246	0.818	0.954	0.136	539.81	571.84	0.806	0.934	0.128	510.57	572.28	0.826	0.914	0.088	551.16	572.90
alpha-d-glucose	3.5219	3.5547	0.964	0.974	0.010	399.81	476.45	0.948	0.962	0.014	385.17	476.24	0.965	0.974	0.009	406.40	476.23
alpha-d-glucose	5.2301	5.2452	0.810	0.996	0.185	317.22	449.19	0.853	0.995	0.142	328.24	449.26	0.818	0.992	0.174	304.15	449.73
glycine	3.5558	3.5658	0.983	0.977	-0.006	1599.48	867.14	0.962	0.947	-0.015	1567.93	867.03	0.993	0.989	-0.004	1657.06	870.09
l-alanine	1.4736	1.4943	0.905	0.990	0.085	3169.67	1370.27	0.878	0.980	0.102	3124.05	1370.43	0.929	0.990	0.061	3168.52	1370.36
l-alanine	3.7645	3.8099	0.703	0.624	-0.079	2285.34	479.71	0.649	0.598	-0.051	2211.41	479.71	0.685	0.649	-0.036	2159.56	479.40
l-arginine	1.8586	1.9571	0.378	0.551	0.173	6573.01	953.51	0.349	0.523	0.174	6367.35	955.21	0.382	0.580	0.198	6618.48	963.12
l-arginine	1.8586	1.9571	0.378	0.551	0.173	6573.01	953.51	0.349	0.523	0.174	6367.35	955.21	0.382	0.580	0.198	6618.48	963.12
l-arginine	1.7030	1.7788	0.792	0.828	0.037	4455.19	489.05	0.784	0.835	0.050	4395.13	489.02	0.795	0.840	0.046	4389.34	489.01
l-arginine	1.6174	1.7023	0.680	0.668	-0.013	1683.14	492.47	0.678	0.668	-0.010	1643.68	492.42	0.689	0.675	-0.014	1667.44	492.46
l-arginine	3.7542	3.7870	0.563	0.678	0.115	2784.71	943.10	0.565	0.723	0.158	2778.36	945.21	0.621	0.807	0.186	2888.39	949.11
l-arginine	3.2290	3.2678	0.479	0.951	0.472	2859.11	478.78	0.435	0.935	0.500	2941.07	478.86	0.467	0.915	0.448	2795.79	478.65
l-asparagine	2.8327	2.8856	0.920	0.915	-0.006	979.40	474.55	0.909	0.908	-0.001	942.94	478.06	0.950	0.952	0.002	943.12	481.75
l-asparagine	2.9256	2.9739	0.426	0.989	0.564	940.01	473.69	0.376	0.975	0.599	894.92	477.81	0.515	0.994	0.479	932.33	480.45
l-asparagine	3.9829	4.0170	0.601	0.751	0.150	3303.24	459.27	0.614	0.727	0.113	3137.54	471.04	0.796	0.750	-0.047	3156.32	475.96
l-glutamic acid	2.3104	2.4005	0.744	0.992	0.248	2483.37	979.47	0.723	0.991	0.268	2440.13	979.43	0.737	0.991	0.254	2486.45	979.35
l-glutamic acid	2.3104	2.4005	0.744	0.992	0.248	2483.37	979.47	0.723	0.991	0.268	2440.13	979.43	0.737	0.991	0.254	2486.45	979.35
l-glutamic acid	2.1021	2.1686	0.493	0.614	0.121	5948.25	488.13	0.457	0.586	0.129	5877.48	488.11	0.478	0.604	0.126	5939.21	487.92
l-glutamic acid	2.0234	2.0974	0.846	0.842	-0.004	2115.56	489.94	0.848	0.845	-0.003	2102.67	490.02	0.862	0.858	-0.005	2115.34	490.28
l-glutamic acid	3.7464	3.7764	0.851	0.845	-0.006	2474.20	471.46	0.817	0.805	-0.011	2564.08	471.32	0.820	0.798	-0.022	2764.42	470.33
l-glutamine	2.4086	2.5052	0.901	0.956	0.056	1915.51	978.81	0.906	0.940	0.034	1873.28	978.82	0.932	0.963	0.031	1917.55	978.74
l-glutamine	2.4086	2.5052	0.901	0.956	0.056	1915.51	978.81	0.906	0.940	0.034	1873.28	978.82	0.932	0.963	0.031	1917.55	978.74
l-glutamine	2.1002	2.1796	0.601	0.432	-0.169	6120.31	976.40	0.556	0.431	-0.125	6042.98	976.38	0.589	0.645	0.056	6165.06	976.00
l-glutamine	2.1002	2.1796	0.601	0.432	-0.169	6120.31	976.40	0.556	0.431	-0.125	6042.98	976.38	0.589	0.645	0.056	6165.06	976.00
l-glutamine	3.7549	3.7881	0.754	0.761	0.007	2794.71	473.02	0.710	0.786	0.076	2773.04	471.60	0.614	0.868	0.255	2876.08	473.97
l-histidine	3.1082	3.1650	0.652	0.635	-0.017	1297.10	3.07	0.658	0.640	-0.018	1456.05	3.54	0.523	0.496	-0.027	1735.30	5.79
l-histidine	3.2197	3.2679	0.171	0.253	0.082	2904.09	24.60	0.181	0.408	0.227	2982.51	19.59	0.242	0.401	0.159	3020.42	17.61
l-histidine	7.0626	7.0834	0.573	0.951	0.378	657.88	466.15	0.570	0.956	0.387	606.95	466.17	0.587	0.945	0.358	795.03	467.37
l-histidine	7.8054	7.8263	0.372	0.399	0.028	4.56	0.79	0.780	0.798	0.018	20.17	2.15	0.608	0.935	0.327	755.19	467.55
l-histidine	3.9715	4.0156	0.541	0.801	0.260	3528.45	479.68	0.512	0.828	0.316	3485.27	480.40	0.601	0.845	0.244	3768.85	481.71
l-isoleucine	0.9263	0.9610	0.891	0.885	-0.006	4588.59	1425.16	0.888	0.885	-0.002	4524.44	1425.17	0.889	0.881	-0.008	4523.60	1425.19
l-isoleucine	1.4397	1.5166	0.466	0.702	0.236	4909.20	487.26	0.441	0.680	0.239	4846.61	487.32	0.491	0.725	0.234	4907.49	487.24
l-isoleucine	1.2251	1.3119	0.894	0.902	0.008	1858.67	488.29	0.879	0.895	0.016	1778.90	488.35	0.898	0.897	-0.001	1904.78	488.30
l-isoleucine	1.9464	2.0248	0.829	0.814	-0.016	2257.87	488.14	0.816	0.805	-0.012	2200.45	488.17	0.827	0.815	-0.011	2227.64	488.12
l-isoleucine	1.0055	1.0247	0.988	0.988	0.000	2837.60	1356.59	0.973	0.983	0.010	2793.80	1356.63	0.985	0.993	0.008	2813.97	1358.15
l-isoleucine	3.6668	3.6865	0.761	0.987	0.227	870.06	461.54	0.799	0.972	0.173	850.86	461.27	0.994	0.995	0.001	881.89	458.62
l-leucine	0.9494	0.9815	0.962	0.961	-0.001	6213.06	2802.77	0.960	0.959	-0.001	6122.86	2802.84	0.963	0.965	0.001	6143.13	2802.70
l-leucine	1.6509	1.7854	0.802	0.860	0.059	5908.31	1465.96	0.801	0.862	0.061	5829.60	1466.05	0.809	0.849	0.040	5831.59	1466.07
l-leucine	0.9494	0.9815	0.962	0.961	-0.001	6213.06	2802.77	0.960	0.959	-0.001	6122.86	2802.84	0.963	0.965	0.001	6143.13	2802.70

l-leucine	1.6509	1.7854	0.802	0.860	0.059	5908.31	1465.96	0.801	0.862	0.061	5829.60	1466.05	0.809	0.849	0.040	5831.59	1466.07
l-leucine	1.6509	1.7854	0.802	0.860	0.059	5908.31	1465.96	0.801	0.862	0.061	5829.60	1466.05	0.809	0.849	0.040	5831.59	1466.07
l-leucine	3.7249	3.7548	0.690	0.873	0.183	1451.81	455.97	0.697	0.847	0.150	1415.04	456.48	0.840	0.898	0.058	1496.26	456.77
l-lysine	3.7452	3.7763	0.745	0.637	-0.109	2485.96	332.27	0.737	0.672	-0.065	2567.29	341.08	0.749	0.735	-0.014	2795.82	355.22
l-lysine	1.8529	1.9568	0.581	0.545	-0.036	6578.38	961.06	0.551	0.526	-0.025	6373.19	961.16	0.605	0.614	0.009	6623.98	961.09
l-lysine	1.8529	1.9568	0.581	0.545	-0.036	6578.38	961.06	0.551	0.526	-0.025	6373.19	961.16	0.605	0.614	0.009	6623.98	961.09
l-lysine	1.4096	1.5606	0.363	0.346	-0.017	5639.74	980.42	0.345	0.329	-0.017	5547.90	980.42	0.377	0.369	-0.008	5645.72	980.56
l-lysine	1.4096	1.5606	0.363	0.346	-0.017	5639.74	980.42	0.345	0.329	-0.017	5547.90	980.42	0.377	0.369	-0.008	5645.72	980.56
l-lysine	1.7012	1.7802	0.785	0.912	0.127	4518.80	969.70	0.788	0.907	0.119	4461.92	969.73	0.798	0.908	0.110	4457.28	969.66
l-lysine	1.7012	1.7802	0.785	0.912	0.127	4518.80	969.70	0.788	0.907	0.119	4461.92	969.73	0.798	0.908	0.110	4457.28	969.66
l-lysine	3.0111	3.0428	0.231	0.662	0.431	4074.28	933.13	0.216	0.649	0.433	4027.71	934.84	0.235	0.661	0.427	4032.61	935.39
l-methionine	2.1015	2.1677	0.944	0.964	0.020	5951.67	1887.66	0.914	0.947	0.033	5883.65	1888.78	0.966	0.971	0.005	5947.32	1889.69
l-methionine	2.1693	2.2390	0.826	0.933	0.107	1203.21	517.71	0.772	0.925	0.153	1153.51	518.82	0.724	0.934	0.210	1207.98	523.22
l-methionine	2.1015	2.1677	0.944	0.964	0.020	5951.67	1887.66	0.914	0.947	0.033	5883.65	1888.78	0.966	0.971	0.005	5947.32	1889.69
l-methionine	3.8339	3.8539	0.839	0.771	-0.068	1297.12	374.37	0.797	0.727	-0.071	1325.04	420.98	0.698	0.749	0.051	1461.50	555.07
l-methionine	3.8474	3.8774	0.482	0.794	0.312	1324.39	738.77	0.467	0.776	0.309	1210.08	696.54	0.555	0.828	0.273	1231.24	607.26
l-methionine	2.6296	2.6653	0.909	0.863	-0.047	3043.69	474.59	0.902	0.847	-0.055	3018.94	474.48	0.923	0.883	-0.040	2974.99	474.61
l-phenylalanine	7.3594	7.3988	0.962	0.965	0.003	871.83	490.57	0.956	0.962	0.007	869.62	490.63	0.978	0.968	-0.010	883.59	490.62
l-phenylalanine	7.4115	7.4508	0.938	0.992	0.054	1669.77	958.83	0.938	0.985	0.047	1662.57	958.81	0.977	0.995	0.018	1705.94	958.70
l-phenylalanine	7.3180	7.3492	0.990	0.973	-0.018	1703.98	950.41	0.983	0.970	-0.013	1680.72	950.41	0.905	0.973	0.068	1726.44	950.65
l-phenylalanine	3.1100	3.1564	0.931	0.895	-0.036	1211.47	475.48	0.931	0.904	-0.027	1229.36	475.77	0.891	0.828	-0.063	1564.34	477.24
l-phenylalanine	3.2679	3.3097	0.846	0.917	0.071	1034.64	475.67	0.944	0.974	0.031	869.86	476.13	0.980	0.974	-0.006	888.74	475.24
l-phenylalanine	3.9859	4.0059	0.873	0.843	-0.030	2325.07	355.01	0.850	0.954	0.104	2197.10	360.72	0.935	0.882	-0.053	2411.53	397.54
l-proline	1.9568	2.1007	0.795	0.845	0.050	4217.73	1453.55	0.797	0.850	0.053	4156.84	1453.34	0.793	0.846	0.054	4208.83	1454.83
l-proline	1.9568	2.1007	0.795	0.845	0.050	4217.73	1453.55	0.797	0.850	0.053	4156.84	1453.34	0.793	0.846	0.054	4208.83	1454.83
l-proline	1.9568	2.1007	0.795	0.845	0.050	4217.73	1453.55	0.797	0.850	0.053	4156.84	1453.34	0.793	0.846	0.054	4208.83	1454.83
l-proline	2.3213	2.3880	0.883	0.911	0.028	2368.08	484.26	0.884	0.899	0.016	2332.86	484.17	0.898	0.925	0.027	2360.94	484.19
l-proline	4.1168	4.1531	0.410	0.461	0.051	785.14	474.45	0.310	0.424	0.114	606.83	474.32	0.817	0.915	0.098	347.42	474.48
l-proline	3.3161	3.3665	0.901	0.992	0.091	700.49	480.91	0.711	0.984	0.273	685.66	480.84	0.698	0.994	0.296	715.06	480.75
l-proline	3.3969	3.4493	0.638	0.763	0.126	1798.83	482.89	0.604	0.773	0.169	1736.65	482.89	0.690	0.821	0.131	1577.51	482.85
l-serine	3.9777	4.0140	0.631	0.700	0.069	3377.63	475.63	0.471	0.796	0.325	3402.19	475.49	0.652	0.784	0.132	3562.31	471.61
l-serine	3.9387	3.9677	0.497	0.984	0.488	766.78	402.67	0.614	0.966	0.352	733.49	401.21	0.984	0.989	0.005	820.17	399.08
l-serine	3.8295	3.8543	0.926	0.931	0.005	1448.34	436.61	0.917	0.925	0.008	1463.65	454.31	0.946	0.943	-0.004	1613.41	465.76
l-threonine	1.3200	1.3463	0.988	0.989	0.002	2872.71	1410.52	0.974	0.978	0.004	2815.21	1410.68	0.994	0.994	0.000	2829.21	1410.58
l-threonine	4.2264	4.2755	0.630	0.858	0.228	1991.96	480.32	0.543	0.869	0.327	1903.45	481.30	0.524	0.892	0.368	2084.21	482.28
l-threonine	3.5747	3.5931	0.825	0.981	0.156	610.54	341.51	0.923	0.952	0.028	748.56	422.64	0.288	0.990	0.703	866.09	457.02
l-valine	1.0299	1.0630	0.561	0.994	0.433	2884.90	1436.79	0.519	0.988	0.469	2830.34	1436.80	0.591	0.996	0.405	2877.36	1436.75
l-valine	0.9826	1.0049	0.934	0.996	0.061	2921.24	1382.15	0.907	0.989	0.082	2886.01	1381.96	0.954	0.996	0.043	2898.05	1384.10
l-valine	2.2379	2.3203	0.830	0.970	0.140	1260.72	488.76	0.797	0.974	0.178	1207.01	488.78	0.828	0.968	0.139	1265.60	488.81
l-valine	3.5992	3.6259	0.757	0.969	0.213	746.89	472.26	0.634	0.940	0.306	721.74	472.44	0.566	0.978	0.412	758.13	472.71

Supplementary Table 5c Cosine improvement scores for GISSMO after remediation for experimental standard mixture at 800 MHz pH 7.2, 7.4 and 7.6, remediation change 3-color scale from -1 to 0 (blue to white) and 0 to +1 (white to red). Metabolite name (name) minimum bound (min), maximal bound (max) Original score (orig), CASMDB score (CASM), difference in score (diff) region integration original x0.00001 (o-int) region integration CASMDB (c-int).

name	min	max	orig	CASM	diff	o-int	c-int	orig	CASM	diff	o-int	c-int	orig	CASM	diff	o-int	c-int
			pH 7.2					pH 7.4					pH 7.6				
citric acid	2.5200	2.5600	0.979	0.959	-0.020	777.38	954.24	0.995	0.988	-0.007	662.65	954.89	0.992	0.980	-0.012	712.65	954.46
citric acid	2.6400	2.6800	0.362	0.814	0.452	1454.02	955.08	0.517	0.682	0.165	1330.32	955.06	0.543	0.769	0.226	1347.42	954.91
creatine	3.0353	3.0436	0.990	0.959	-0.031	1151.43	1258.98	0.989	0.961	-0.029	1124.95	1258.97	0.993	0.970	-0.023	1100.04	1258.56
creatine	3.9278	3.9373	0.099	0.954	0.855	666.64	861.02	0.104	0.956	0.852	656.69	861.02	0.164	0.968	0.804	674.38	860.66
creatinine	3.0430	3.0510	0.925	0.898	-0.027	1003.56	1245.42	0.931	0.898	-0.033	982.63	1243.21	0.953	0.928	-0.025	977.27	1237.60
creatinine	4.0500	4.0600	0.679	0.800	0.121	73.71	859.49	0.644	0.775	0.131	72.67	856.23	0.642	0.751	0.109	65.29	850.01
alpha-d-glucose	4.6395	4.6621	0.265	0.879	0.613	188.16	461.79	0.240	0.884	0.645	184.69	461.96	0.268	0.923	0.655	202.53	462.03
alpha-d-glucose	3.8822	3.9161	0.653	0.829	0.177	547.51	476.92	0.662	0.830	0.168	542.40	476.93	0.650	0.878	0.228	574.11	476.91
alpha-d-glucose	3.7247	3.7456	0.785	0.882	0.097	385.65	393.40	0.739	0.886	0.147	384.97	397.71	0.731	0.864	0.134	411.23	400.23
alpha-d-glucose	3.4519	3.4890	0.827	0.908	0.082	311.12	635.81	0.837	0.906	0.069	300.49	634.65	0.879	0.947	0.069	308.05	634.22
alpha-d-glucose	3.3871	3.4104	0.837	0.832	-0.005	471.50	422.08	0.884	0.888	0.004	389.17	420.50	0.851	0.845	-0.006	376.87	413.98
alpha-d-glucose	3.4764	3.5153	0.868	0.892	0.023	282.66	590.09	0.872	0.896	0.024	281.94	590.15	0.908	0.922	0.014	288.20	591.82
alpha-d-glucose	3.2300	3.2681	0.776	0.807	0.032	1270.86	476.78	0.695	0.734	0.040	1279.85	476.67	0.620	0.670	0.050	1206.43	476.53
alpha-d-glucose	3.7464	3.7875	0.766	0.779	0.013	1535.90	492.23	0.770	0.807	0.038	1544.96	492.49	0.657	0.810	0.153	1509.26	491.92
alpha-d-glucose	3.8194	3.8666	0.626	0.660	0.034	957.58	967.33	0.677	0.624	-0.052	996.71	967.32	0.676	0.748	0.072	994.54	967.48
alpha-d-glucose	3.8194	3.8666	0.626	0.660	0.034	957.58	967.33	0.677	0.624	-0.052	996.71	967.32	0.676	0.748	0.072	994.54	967.48
alpha-d-glucose	3.4109	3.4379	0.894	0.929	0.036	410.45	539.26	0.902	0.919	0.017	402.35	540.40	0.917	0.872	-0.044	410.29	545.92
alpha-d-glucose	3.6998	3.7246	0.715	0.910	0.194	180.12	568.12	0.705	0.914	0.209	172.13	562.77	0.733	0.938	0.204	201.22	555.96
alpha-d-glucose	3.5219	3.5547	0.891	0.868	-0.023	134.81	478.24	0.899	0.886	-0.013	136.31	478.13	0.927	0.924	-0.003	152.94	478.13
alpha-d-glucose	5.2301	5.2452	0.805	0.975	0.170	133.36	450.46	0.800	0.980	0.180	132.65	450.53	0.771	0.983	0.212	126.05	450.50
glycine	3.5558	3.5658	0.934	0.863	-0.071	681.51	865.96	0.941	0.880	-0.061	692.66	866.61	0.962	0.909	-0.054	701.99	867.99
l-alanine	1.4736	1.4943	0.846	0.941	0.095	1375.12	1377.87	0.851	0.939	0.088	1350.27	1377.86	0.873	0.956	0.082	1322.91	1377.96
l-alanine	3.7645	3.8099	0.640	0.582	-0.058	1156.75	481.21	0.616	0.584	-0.033	1165.80	481.17	0.624	0.644	0.020	1034.67	480.83
l-arginine	1.8586	1.9571	0.320	0.468	0.148	3004.71	970.77	0.330	0.476	0.145	2855.97	970.50	0.344	0.506	0.162	2960.30	972.37
l-arginine	1.8586	1.9571	0.320	0.468	0.148	3004.71	970.77	0.330	0.476	0.145	2855.97	970.50	0.344	0.506	0.162	2960.30	972.37
l-arginine	1.7030	1.7788	0.751	0.857	0.107	1963.47	489.59	0.758	0.861	0.103	1921.01	489.61	0.773	0.876	0.103	1892.79	489.66
l-arginine	1.6174	1.7023	0.677	0.667	-0.010	855.26	492.56	0.676	0.666	-0.010	828.68	492.54	0.681	0.672	-0.009	785.10	492.58
l-arginine	3.7542	3.7870	0.596	0.712	0.116	1374.47	945.96	0.645	0.782	0.137	1367.24	950.44	0.558	0.755	0.197	1312.32	951.42
l-arginine	3.2290	3.2678	0.438	0.950	0.513	1270.31	479.59	0.401	0.946	0.545	1280.28	479.57	0.425	0.954	0.529	1231.96	479.52
l-asparagine	2.8327	2.8856	0.945	0.923	-0.022	408.79	478.47	0.954	0.926	-0.029	403.44	480.69	0.968	0.949	-0.020	401.09	483.27
l-asparagine	2.9256	2.9739	0.310	0.927	0.617	442.15	479.61	0.332	0.935	0.603	424.46	480.95	0.522	0.955	0.432	426.84	482.06
l-asparagine	3.9829	4.0170	0.566	0.672	0.106	1434.09	467.23	0.570	0.688	0.118	1471.33	473.40	0.592	0.697	0.106	1317.74	476.77
l-glutamic acid	2.3104	2.4005	0.494	0.969	0.475	1294.67	979.94	0.523	0.972	0.449	1235.13	979.85	0.520	0.969	0.449	1230.37	979.77
l-glutamic acid	2.3104	2.4005	0.494	0.969	0.475	1294.67	979.94	0.523	0.972	0.449	1235.13	979.85	0.520	0.969	0.449	1230.37	979.77
l-glutamic acid	2.1021	2.1686	0.424	0.470	0.046	2306.78	488.80	0.404	0.467	0.063	2328.79	488.81	0.372	0.519	0.147	2209.05	488.71
l-glutamic acid	2.0234	2.0974	0.868	0.879	0.011	1085.78	490.56	0.863	0.876	0.013	1059.76	490.57	0.882	0.890	0.008	1063.73	490.73
l-glutamic acid	3.7464	3.7764	0.780	0.805	0.025	1105.85	472.85	0.791	0.793	0.001	1151.22	472.81	0.743	0.764	0.021	1222.05	471.87
l-glutamine	2.4086	2.5052	0.834	0.873	0.039	794.27	979.42	0.838	0.874	0.036	779.56	979.38	0.831	0.881	0.050	751.27	979.34
l-glutamine	2.4086	2.5052	0.834	0.873	0.039	794.27	979.42	0.838	0.874	0.036	779.56	979.38	0.831	0.881	0.050	751.27	979.34
l-glutamine	2.1002	2.1796	0.524	0.438	-0.085	2365.50	976.88	0.519	0.467	-0.051	2388.56	976.65	0.547	0.694	0.147	2271.05	976.28
l-glutamine	2.1002	2.1796	0.524	0.438	-0.085	2365.50	976.88	0.519	0.467	-0.051	2388.56	976.65	0.547	0.694	0.147	2271.05	976.28
l-glutamine	3.7549	3.7881	0.733	0.749	0.016	1348.60	473.37	0.698	0.802	0.105	1352.85	473.25	0.605	0.833	0.228	1306.99	474.97
l-histidine	3.1082	3.1650	0.611	0.596	-0.016	579.92	2.95	0.549	0.533	-0.016	626.56	3.41	0.506	0.479	-0.027	727.54	5.21
l-histidine	3.2197	3.2679	0.208	0.296	0.088	1281.91	18.64	0.078	0.396	0.319	1292.15	16.00	0.145	0.409	0.264	1280.94	14.08
l-histidine	7.0626	7.0834	0.561	0.946	0.385	281.59	465.67	0.607	0.933	0.326	331.78	465.67	0.608	0.923	0.316	327.25	466.90
l-histidine	7.8054	7.8263	0.560	0.537	-0.023	4.37	0.76	0.237	0.230	-0.007	1.45	2.03	0.585	0.962	0.377	237.06	467.09
l-histidine	3.9715	4.0156	0.493	0.728	0.235	1478.76	482.33	0.509	0.714	0.204	1533.26	482.17	0.596	0.870	0.274	1501.27	482.23
l-isoleucine	0.9263	0.9610	0.834	0.814	-0.020	1998.22	1430.12	0.839	0.825	-0.014	1951.73	1430.83	0.839	0.826	-0.014	1863.01	1430.05
l-isoleucine	1.4397	1.5166	0.372	0.471	0.100	2141.74	487.92	0.374	0.484	0.111	2088.58	487.92	0.397	0.510	0.114	2055.80	487.87
l-isoleucine	1.2251	1.3119	0.811	0.860	0.049	764.49	488.85	0.821	0.865	0.044	741.90	488.84	0.840	0.873	0.033	759.66	488.82
l-isoleucine	1.9464	2.0248	0.757	0.746	-0.011	1007.55	488.48	0.755	0.747	-0.008	991.17	488.52	0.770	0.767	-0.003	1022.69	488.49
l-isoleucine	1.0055	1.0247	0.961	0.945	-0.016	1299.76	1366.07	0.956	0.948	-0.008	1272.64	1366.06	0.961	0.960	-0.001	1253.01	1365.61
l-isoleucine	3.6668	3.6865	0.675	0.919	0.244	358.71	462.38	0.797	0.916	0.119	358.81	461.89	0.940	0.949	0.009	372.21	459.65
l-leucine	0.9494	0.9815	0.924	0.931	0.006	2774.64	2814.06	0.924	0.937	0.012	2713.55	2813.95	0.940	0.945	0.005	2659.60	2814.10
l-leucine	1.6509	1.7854	0.770	0.784	0.014	2702.14	1466.52	0.776	0.787	0.011	2638.79	1466.66	0.781	0.783	0.003	2576.21	1466.42

l-leucine	0.9494	0.9815	0.924	0.931	0.006	2774.64	2814.06	0.924	0.937	0.012	2713.55	2813.95	0.940	0.945	0.005	2659.60	2814.10
l-leucine	1.6509	1.7854	0.770	0.784	0.014	2702.14	1466.52	0.776	0.787	0.011	2638.79	1466.66	0.781	0.783	0.003	2576.21	1466.42
l-leucine	1.6509	1.7854	0.770	0.784	0.014	2702.14	1466.52	0.776	0.787	0.011	2638.79	1466.66	0.781	0.783	0.003	2576.21	1466.42
l-leucine	3.7249	3.7548	0.568	0.713	0.144	572.59	461.36	0.604	0.704	0.100	578.84	461.20	0.682	0.726	0.044	623.67	460.80
l-lysine	3.7452	3.7763	0.773	0.641	-0.132	1112.23	329.16	0.814	0.649	-0.165	1157.71	342.58	0.665	0.703	0.038	1238.69	363.18
l-lysine	1.8529	1.9568	0.298	0.477	0.180	3008.96	973.11	0.322	0.434	0.112	2860.03	972.60	0.339	0.445	0.105	2961.56	973.27
l-lysine	1.8529	1.9568	0.298	0.477	0.180	3008.96	973.11	0.322	0.434	0.112	2860.03	972.60	0.339	0.445	0.105	2961.56	973.27
l-lysine	1.4096	1.5606	0.280	0.300	0.020	2510.47	981.94	0.278	0.295	0.018	2448.43	981.92	0.289	0.305	0.016	2409.98	981.92
l-lysine	1.4096	1.5606	0.280	0.300	0.020	2510.47	981.94	0.278	0.295	0.018	2448.43	981.92	0.289	0.305	0.016	2409.98	981.92
l-lysine	1.7012	1.7802	0.810	0.928	0.117	2009.68	974.54	0.812	0.929	0.117	1966.85	974.49	0.825	0.927	0.103	1932.56	974.15
l-lysine	1.7012	1.7802	0.810	0.928	0.117	2009.68	974.54	0.812	0.929	0.117	1966.85	974.49	0.825	0.927	0.103	1932.56	974.15
l-lysine	3.0111	3.0428	0.259	0.501	0.243	1786.10	942.59	0.243	0.506	0.263	1740.40	942.60	0.260	0.509	0.249	1701.17	943.29
l-methionine	2.1015	2.1677	0.885	0.894	0.009	2303.18	1906.82	0.894	0.906	0.011	2324.93	1911.06	0.919	0.920	0.001	2205.40	1926.52
l-methionine	2.1693	2.2390	0.756	0.860	0.104	529.85	496.05	0.737	0.895	0.159	544.74	497.20	0.687	0.909	0.222	514.60	500.90
l-methionine	2.1015	2.1677	0.885	0.894	0.009	2303.18	1906.82	0.894	0.906	0.011	2324.93	1911.06	0.919	0.920	0.001	2205.40	1926.52
l-methionine	3.8339	3.8539	0.743	0.862	0.119	589.33	824.85	0.703	0.834	0.131	615.58	825.06	0.764	0.827	0.062	664.84	828.35
l-methionine	3.8474	3.8774	0.513	0.712	0.199	672.64	323.34	0.547	0.685	0.138	647.72	284.44	0.650	0.615	-0.036	627.96	312.22
l-methionine	2.6296	2.6653	0.785	0.754	-0.031	1203.81	476.09	0.884	0.867	-0.017	1139.04	476.08	0.776	0.809	0.032	1119.08	476.05
l-phenylalanine	7.3594	7.3988	0.933	0.964	0.032	366.25	490.75	0.954	0.971	0.017	376.66	490.75	0.960	0.974	0.014	371.83	490.75
l-phenylalanine	7.4115	7.4508	0.889	0.978	0.088	705.62	960.95	0.922	0.979	0.058	715.29	960.94	0.959	0.988	0.029	705.96	960.79
l-phenylalanine	7.3180	7.3492	0.982	0.974	-0.008	722.05	952.10	0.976	0.975	-0.001	732.82	952.10	0.870	0.978	0.108	718.96	952.20
l-phenylalanine	3.1100	3.1564	0.930	0.939	0.009	518.38	478.71	0.955	0.975	0.020	540.82	478.97	0.897	0.870	-0.027	689.21	479.65
l-phenylalanine	3.2679	3.3097	0.745	0.885	0.139	459.91	478.14	0.900	0.956	0.056	385.61	478.20	0.971	0.970	-0.001	396.28	477.57
l-phenylalanine	3.9859	4.0059	0.848	0.781	-0.067	1038.33	366.95	0.839	0.882	0.042	1010.77	376.47	0.935	0.936	0.001	1029.45	436.01
l-proline	1.9568	2.1007	0.778	0.867	0.089	2047.19	1469.34	0.774	0.871	0.097	2001.43	1469.53	0.765	0.878	0.113	2029.04	1469.68
l-proline	1.9568	2.1007	0.778	0.867	0.089	2047.19	1469.34	0.774	0.871	0.097	2001.43	1469.53	0.765	0.878	0.113	2029.04	1469.68
l-proline	1.9568	2.1007	0.778	0.867	0.089	2047.19	1469.34	0.774	0.871	0.097	2001.43	1469.53	0.765	0.878	0.113	2029.04	1469.68
l-proline	2.3213	2.3880	0.900	0.938	0.038	1175.00	485.90	0.900	0.940	0.040	1124.97	485.89	0.906	0.949	0.043	1113.47	485.87
l-proline	4.1168	4.1531	0.485	0.659	0.174	447.45	476.44	0.456	0.618	0.161	396.50	476.39	0.843	0.967	0.124	247.68	476.44
l-proline	3.3161	3.3665	0.778	0.959	0.182	331.45	482.76	0.642	0.965	0.323	333.53	482.72	0.537	0.979	0.442	337.67	482.75
l-proline	3.3969	3.4493	0.570	0.746	0.176	797.92	484.22	0.551	0.776	0.225	771.17	484.21	0.604	0.842	0.237	652.78	484.14
l-serine	3.9777	4.0140	0.572	0.783	0.211	1422.29	477.89	0.513	0.758	0.245	1510.30	477.90	0.572	0.815	0.243	1457.99	475.26
l-serine	3.9387	3.9677	0.410	0.918	0.508	390.28	424.96	0.583	0.929	0.346	369.31	416.40	0.936	0.945	0.009	328.45	402.20
l-serine	3.8295	3.8543	0.889	0.881	-0.008	628.41	450.93	0.910	0.905	-0.005	653.31	459.63	0.918	0.914	-0.005	706.80	467.38
l-threonine	1.3200	1.3463	0.955	0.933	-0.022	1200.30	1413.35	0.967	0.927	-0.040	1167.91	1413.55	0.980	0.947	-0.033	1146.15	1412.45
l-threonine	4.2264	4.2755	0.503	0.811	0.309	818.34	482.09	0.450	0.835	0.386	820.81	482.57	0.510	0.863	0.353	824.26	483.10
l-threonine	3.5747	3.5931	0.736	0.878	0.143	279.80	380.82	0.860	0.889	0.030	342.97	433.34	0.235	0.919	0.684	358.85	458.14
l-valine	1.0299	1.0630	0.455	0.948	0.493	1283.00	1437.82	0.459	0.955	0.495	1253.38	1437.81	0.507	0.973	0.466	1248.04	1437.77
l-valine	0.9826	1.0049	0.873	0.951	0.078	1273.85	1388.87	0.879	0.953	0.073	1241.13	1388.91	0.906	0.970	0.063	1227.22	1390.31
l-valine	2.2379	2.3203	0.794	0.947	0.152	652.75	489.21	0.809	0.962	0.154	636.08	489.22	0.835	0.944	0.110	674.37	489.20
l-valine	3.5992	3.6259	0.662	0.891	0.229	350.08	472.50	0.615	0.895	0.281	349.23	472.65	0.514	0.925	0.411	352.86	472.93
